# Pivotal trial of an autonomous AI-based diagnostic system for detection of diabetic retinopathy in primary care offices

**DOI:** 10.1038/s41746-018-0040-6

**Published:** 2018-08-28

**Authors:** Michael D. Abràmoff, Philip T. Lavin, Michele Birch, Nilay Shah, James C. Folk

**Affiliations:** 1 Department of Ophthalmology and Visual Sciences, University of Iowa, Iowa City, IA 52242 USA; 2 Veterans Administration Medical Center, Iowa City, IA 52242 USA; 3 IDx LLC, Coralville, IA 52241 USA; 4 Institute for Vision Research, University of Iowa, Iowa City, IA 52242 USA; 5 Boston Biostatistics Research Foundation, Inc., 3 Cahill Park Drive, Framingham, MA 01702 USA; 6 Department of Family Medicine, Director of Academic Services, University of North Carolina School of Medicine, Charlotte, NC 28204 USA; 7 The Emmes Corporation, 401 North Washington Street, Suite 700, Rockville, MD 20850 USA

**Keywords:** Eye manifestations, Biomedical engineering, Eye manifestations, Biomedical engineering

## Abstract

Artificial Intelligence (AI) has long promised to increase healthcare affordability, quality and accessibility but FDA, until recently, had never authorized an autonomous AI diagnostic system. This pivotal trial of an AI system to detect diabetic retinopathy (DR) in people with diabetes enrolled 900 subjects, with no history of DR at primary care clinics, by comparing to Wisconsin Fundus Photograph Reading Center (FPRC) widefield stereoscopic photography and macular Optical Coherence Tomography (OCT), by FPRC certified photographers, and FPRC grading of Early Treatment Diabetic Retinopathy Study Severity Scale (ETDRS) and Diabetic Macular Edema (DME). More than mild DR (mtmDR) was defined as ETDRS level 35 or higher, and/or DME, in at least one eye. AI system operators underwent a standardized training protocol before study start. Median age was 59 years (range, 22–84 years); among participants, 47.5% of participants were male; 16.1% were Hispanic, 83.3% not Hispanic; 28.6% African American and 63.4% were not; 198 (23.8%) had mtmDR. The AI system exceeded all pre-specified superiority endpoints at sensitivity of 87.2% (95% CI, 81.8–91.2%) (>85%), specificity of 90.7% (95% CI, 88.3–92.7%) (>82.5%), and imageability rate of 96.1% (95% CI, 94.6–97.3%), demonstrating AI’s ability to bring specialty-level diagnostics to primary care settings. Based on these results, FDA authorized the system for use by health care providers to detect more than mild DR and diabetic macular edema, making it, the first FDA authorized autonomous AI diagnostic system in any field of medicine, with the potential to help prevent vision loss in thousands of people with diabetes annually. ClinicalTrials.gov NCT02963441

## Introduction

People with diabetes fear visual loss and blindness more than any other complication.^1^ Diabetic retinopathy (DR) is the primary cause of blindness and visual loss among working age men and women in the United States and causes more than 24,000 people to lose vision each year.^2,3^ Adherence to regular eye examinations is necessary to diagnose DR at an early stage, when it can be treated with the best prognosis,^4,5^ and have resulted in substantial reductions in visual loss and blindness.^6^ Despite this, less than 50% of patients with diabetes adhere to the recommended schedule of eye exams,^7^ and adherence has not increased over the last 15 years despite large-scale efforts to increase it.^8^ To increase adherence, retinal imaging in or close to primary care offices followed by remote evaluation using telemedicine has also been widely studied.^9–11^

Artificial intelligence (AI)-based algorithms to detect DR from retinal images have been examined in laboratory settings.^12–15^ Recent advances incorporate improved machine learning into these algorithms have led to higher diagnostic accuracy.^16,17^ However, in addition to high diagnostic accuracy, responsible and safe implementation in primary care requires autonomy (i.e., a use case that removes the requirement for review by human experts), instantaneous image quality feedback to the primary care based operator in order to reach a reliable disease level output in the vast majority of patients, a realtime clinical decision at the point of care, and consistent diagnostic accuracy across age, race and ethnicity.^12,13,18,19^ Studies comparing an AI system against an independent, high-quality gold standard that includes fundus imaging and Optical Coherence Tomography (OCT) imaging protocols have not previously been conducted; FDA has not previously authorized any such system.

The Wisconsin Fundus Photograph Reading Center (FPRC) has historically been the gold standard for trials that require grading of the severity of DR, including the Epidemiology of Diabetes Interventions and Complications/Diabetes Control and Complications Trial (EDIC/DCCT), Diabetic Retinopathy Clinical Research Network (DRCR.net) studies, as well as pivotal phase III studies.^20,21^ The FPRC has adopted the use of a widefield stereoscopic retinal imaging protocol (4W-D), that includes four stereoscopic pairs of digital images per eye, each pair covering 45–60°, equivalent to the area of the retina covered by the older, modified 7-field stereo film protocol.^22,23^ Traditionally, the presence of Diabetic Macular Edema (DME) was determined from the stereo fundus photos, but more recently, Optical Coherence Tomography (OCT) has become the reference modality for determining the presence or absence of center-involved DME.^24^ The 2017 revision of the American Academy of Ophthalmology’s Preferred Practice Pattern recommends people with no or mild DR be followed annually, whereas those with more than mild DR, and/or DME (abbreviated to mtmDR), are recommended to receive evaluation and consideration for treatment.^25,26^

In this study, we evaluate the diagnostic performance of an autonomous AI system for the automated detection of DR and DME, termed mtmDR. Study subjects were people with diabetes, not previously known to have DR or DME, under an intent-to-screen (ITS) study design. Ten study sites were all in primary care offices, and the AI systems were operated by existing staff at those sites, using standardized training and operator materials to facilitate use of the system. The FPRC imaging on the other hand was conducted by FPRC certified expert photographers.

## Results

### Study population

**Fig. 1:**
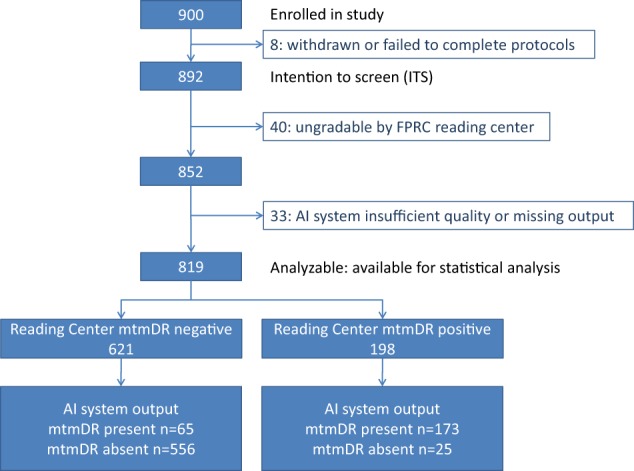
Waterfall diagram showing the final disposition of each participant in the enrolled, intention to screen (ITS), and fully analyzable populations

A total of 900 participants were enrolled at 10 sites, of which 892 participants completed all procedures. A subset of 819 of these participants could be fully analyzed, see Fig. 1, giving an analyzable fraction of 92% (95% CI, 90%–93%). Median age was 59 years (range, 22–84 years); 47.5% of participants were male. For the entire group of participants 16.1% were Hispanic, 83.3% were not Hispanic, and 0.6% unknown. Also, 63.4% were white, 28.6% African American, and 1.6% Asian (Table 1). Finally 7.1% had type 1 diabetes, 92.9% had type 2 diabetes. The 819 participants whose results could be fully evaluated and the 73 participants whose results could not be analyzed, differed significantly with respect to lens status, while mean age, ethnicity, race, and HbA1C level were not significantly different (Table 1). The enrichment strategy led to 319 enriched participants; the 621 No/Mild DR participants included 218 participants from enrichment while the 198 mtmDR participants included 101 participants from enrichment. In the subset of participants enrolled without enrichment, mean HbA1C ± std was 8.1 ± 1.6 mmol/l, while for all participants overall mean HbA1C ± std was 9.4 ± 2.3 mmol/l. According to the FPRC, 11/819 subjects had “enlarged cup to disc ratio”, and 26/819 subjects had “any drusen” and/or “any retinal pigment epithelium atrophy”—none of these subjects had increased retinal thickness on OCT.

**Table 1:** Demographic characteristics of the analyzable (*n* = 819) and non-analyzable (*n* = 73) ITS subsets

Category	Subgroup	Analyzable % (*n*/*N*)	Not analyzable % (*n*/*N*)
Age (years)	<65	69.1% (566/819)	52.1% (38/73)
	≥65	30.9% (253/819)	47.9% (35/73)
Ethnicity	Hispanic or Latino	16.4% (134/819)	13.7% (10/73)
	Not Hispanic or Latino	83.0% (680/819)	80.8% (59/73)
	Unknown	0.6% (5/819)	5.5% (4/73)
HbA1c7	<7	11.6% (95/819)	23.3% (17/73)
	≥7	86.7% (710/819)	76.7% (56/73)
	Unknown^*^	1.7% (14/819)	0% (0/73)
HbA1c9	<9	46.4% (380/819)	54.8% (40/73)
	≥9	51.9% (425/819)	45.2% (33/73)
	Unknown^*^	1.7% (14/819)	0% (0/73)
Lens Status	Phakic with opacities or Cannot Grade	10.4% (85/819)	47.9% (35/73)
	Pseudophakic or no opacities	89.6% (734/819)	52.1% (38/73)
Race	American Indian or Alaskan Native	0.4% (3/819)	0.0% (0/73)
	Asian	1.5% (12/819)	4.1% (3/73)
	Black	28.2% (231/819)	46.6% (34/73)
	Mixed Race	1.2% (10/819)	1.4% (1/73)
	Other-Mexican	0.1% (1/819)	0.0% (0/73)
	Other-Puerto rican	0.1% (1/819)	0.0% (0/73)
	Refuse to provide	1.1% (9/819)	0.0% (0/73)
	Unknown	3.5% (29/819)	1.4% (1/73)
	White	63.9% (523/819)	46.6% (34/73)

* These subjects had diabetes diagnosed by means other than HbA1C – see Methods

A total of 198 mtmDR participants were fully analyzable according to the FPRC reading protocol, thus prevalence of mtmDR was 23.8% (198/819). Of these, twenty-nine had CSDME according to fundus photography; 19 participants had center-involved DME according to OCT; and 42 participants had either CSDME and/or center-involved DME, with corresponding prevalences of 3.5% for CSDME, 2.3% for center-involved DME, and 5.1% for any DME according to both of these assessments. Average centerfield thickness was 239 µm (±0.05 µm) in the participants with CSDME (from fundus photographs only), and 304 µm (±0.06 µm) in the participants with center-involved OCT DME. See Supplemental Table 1 for the DR frequency distribution according to ETDRS severity levels and to DME.

### AI system characteristics

The AI system correctly identified 173 of the 198 fully analyzable participants with fundus mtmDR. Logistic regression yielded a primary sensitivity of 87.2% (95% CI, 81.8%–91.2%) to fundus mtmDR, and 85.9% (95%% CI, 82.5%–88.7%) to multimodal mtmDR. Observed sensitivity to fundus mtmDR was 87.4% (97.5% CI lower bound, 81.1%) (173/198). The logistic regression model did not identify any significant effects for age, sex, race, ethnicity, HbA1C, lens status, or site, on sensitivity. The retrospective power was 93%. The AI system had a sensitivity to fundus vtDR of 97.4% (95% CI 86.2%–99.9%) (37/38), and to multimodal vtDR of 92.2% (95% CI 81.1%–97.8%) (47/51). Among these, it identified 28 of 29 (96.6%; 95% CI, 82.2%–99.9%) participants with CSDME (fundus photographs only), 16 of 19 participants (84.2%; 95% CI, 60.4%–96.6%) with center-involved DME (OCT only), and all participants with ETDRS level 43 or higher (including all 16 subjects with proliferative DR), with an *mtmDR detected* output.

Among the 621 fully analyzable participants who did not have fundus mtmDR according to FPRC grading, there were 556 participants with an *mtmDR not detected* output. Logistic regression yielded a primary specificity of 90.7% (95% CI, 88.3%–92.7%) for fundus mtmDR, and yielded 90.7% (95% CI, 86.8%–93.5%) specificity for multimodal mtmDR, both after correction for enrichment. Observed specificity to fundus mtmDR was 556/621 or 89.5% (97.5% CI lower bound, 86.5%). A logistic regression model did not identify any significant effects of sex, ethnicity, race, HbA1C, lens status, or site, on specificity, while increased specificity was observed in subjects over 65 years of age (*p* = 0.030). The retrospective power was 87%. See Table 2.

**Table 2:** AI system diagnostic accuracy

	Point estimate	95% CI	Superiority endpoint
Sensitivity	87.2%	81.8%–91.2%	85.0%
Specificity	90.7%	88.3%–92.7%	82.5%

Point estimates for sensitivity and specificity were calculated on the 819 participants that were analyzable, using the prespecified logistic regression. The superiority endpoints were previously discussed with FDA.

Among the 73/892 non-analyzable participants, 40 (4%) lacked a completed FPRC grading. In the worst case scenario (forcing all 40 subjects to a grading that is the opposite of the AI-system, in other words, the FPRC grading was set at mtmDR + if the AI system output was *mtmDR not detected* and vice versa), the sensitivity and specificity would have been 80.7% (two-sided 95% CI, 76.7%–84.2%) and 89.8% (two-sided 95% CI, 85.9%–92.7%) respectively. These results would still rule out the pre-specified inferiority hypotheses.

Of the 852 participants that received a completed FPRC grading, 33 participants (4%) received an *insufficient image quality* output from the AI system after completion of the AI system protocol. Thus image-ability, defined as the percentage of participants with a completed FPRC grading and with a disease level AI system output, was 819/852 (96.1%; 95% CI, 94.6–97.28%). In the 33 participants with AI system insufficient image quality, the prevalence of mtmDR was 10/33 (30%), comparable to the mtmDR prevalence in the fully analyzable dataset. For the AI system protocol, 76.4% of participants did not require pharmacologic dilation, while 23.6% required dilation to obtain an AI system disease level output. The majority of participants, 64.7%, completed the AI system protocol of 4 photographs the first time, 8.5% were able to complete the protocol after a single retry, 3.2% needed 2, 19.7% needed 3, 3.4% needed 4 and 0.5% needed five retries. There were 5/11 subjects with enlarged optic disc cups, and 13/26 subjects with any drusen or RPE atrophy, received an “*mtmDR detected*” output.

## Discussion

The results of this study show that the AI system in a primary care setting robustly exceeded the pre-specified primary endpoint goals with a sensitivity of 87.2% (>85%), a specificity of 90.7% (>82.5%), and an imageability rate of 96.1%. Sensitivity is a patient safety criterion, because the AI system’s primary role is to identify those people with diabetes who are likely to have DR that requires further evaluation by an eye care provider. Previous studies have shown that board-certified ophthalmologists that perform indirect ophthalmoscopy achieve an average sensitivity of 33%,^27^ 34%,^28^ or 73%^9^ compared to the same ETDRS standard.

Specificity is also an important consideration, because it affects the number of people with diabetes who receive a referral but do not need one because they have only no or mild DR. Because all referrals will be evaluated by an eye care provider however, this will not increase the risk of that person receiving unnecessary treatment. The American Academy of Ophthalmology Preferred Practice Pattern (PPP 2017 revision) recommends that people with no or mild DR (ETDRS levels 10–20 and no DME) are followed annually, those with moderate DR (ETDRS level 35–47), and no DME receive more frequent follow-up, and those with more than moderate DR (level 53 or higher), or DME receive immediate evaluation.^26^ The AI system had a sensitivity of 97.6% in identifying people that require immediate evaluation according to the PPP. Primary care providers may not feel comfortable evaluating the retina of a person with diabetes themselves. In that context, an autonomous—i.e., without human expert reading of the retinal images—AI system is helpful if it can identify those people who should receive referral to an eye care provider.

To our knowledge, the severity of DR and diabetic macular edema according to the ETDRS severity scale and OCT in a primary care diabetes population has not been determined previously. The only available studies reported on participants who were followed for some type of intervention, had some level of DR at inclusion, or did not have an ETDRS severity scale reading by a reading center.^29^^–^^31^ In the fully analyzable sample in this study, age, sex, ethnicity, and racial distribution were representative of the US diabetes population,^7^ and the prevalence of mtmDR in this representative sample was 23.8%.

Additionally, the study yielded two reading center based gradings for DME: 1) CSDME, based on fundus photographs, and 2) center-involved DME, based on OCT. The prevalence of DME measured accordingly was lower than typically reported at 3.5% for CSDME, and 2.3% for center-involved DME. Previous studies reported that the majority of DME detected on OCT was detectable by fundus photography, but of the 19 cases in this study that had center-involved DME, only 6 (32%) were identified as such by the FPRC reading center from fundus photographs. These results confirm an earlier report that fundus photography may be underestimating the prevalence and incidence of DME in people with diabetes, compared to OCT.^32^ Nevertheless, 84% of all cases with center-involved DME were detected by the AI system. Sensitivity and specificity met endpoints for *fundus mtmDR*, defined as ETDRS level ≥35, or having CSDME, or both, all determined from fundus photographs only. It also met sensitivity and specificity endpoints for *multimodal mtmDR*, defined as ETDRS level ≥35 (determined from fundus photographs), or having CSDME (determined from fundus photographs), or having center-involved DME (determined from OCT), or any combination of these. All cases of the most severe forms of DR, including proliferative DR, were detected.

While there is widespread evidence for the effectiveness and cost-effectiveness of early detection of DR,^33^ this is presently not the case for glaucoma,^34^ macular degeneration^35^ and many other eye diseases, and thus the present study was not designed or powered to analyze diagnostic accuracy on other retinal abnormalities in people with diabetes. However, we observe the following about so-called incidental findings: 6/819 subjects with enlarged optic disc cups were not flagged by the AI system. Of these, an estimated 33% will have some form of glaucoma.^36^ Thus, ~2/819 subjects (~0.2%) would not have been referred to an eye care provider for disease while possibly having some form of glaucoma. Similarly, 13 subjects with drusen or RPE atrophy, all without retinal thickening, were not flagged, so 13/819 subjects (1.6%), would not have been referred to an eye care provider—some of these with non-exudative age-related macular degeneration and intermediate drusen warranting preventative supplements.^37^ We wish to emphasize that these are observations only, given that the 95% confidence intervals around their estimates include 0%.

As expected, sensitivity of the AI system was lower than that of the almost identical AI system tested on a laboratory dataset, which found a sensitivity of 97%.^16^ Sources for the lower sensitivity are likely to be:

- The use of stereo widefield photography, covering an area of the retina more than twice the size of that imaged for analysis by the AI system. Stereo photography allows CSDME to be estimated even in the absence of any other retinal lesions such as exudates. In the case of the laboratory study, the reference standard was determined from the same non-stereo images as available to the AI system, which does not allow the expert readers to estimate DME in the absence of exudates;
- The use of experienced ophthalmic photographers to obtain the reference standard stereo widefield photographs. This results in an overall higher image quality than that obtained by the primary clinic staff after 4 h of training that was available to the AI system;
- The prospective, ITS paradigm used in this study reduced selection and spectrum bias compared to laboratory studies.

The AI system is “physiologically plausible” to some degree, given its multiple, redundant, lesion-specific detectors for biomarkers,^38^ leading to increased robustness to small perturbations in input images,^39^ and because the biomarkers are based on over a century of worldwide clinical experience, lower expected risk of ethnic or racial bias.^40^ And in fact, diagnostic accuracy of the AI system was robust to sex, race, ethnicity, lens status and metabolic control, though specificity was higher in those over age 65. This is likely related to the prevalence of highly reflective internal limiting membrane in younger people, which can be mistaken for exudates due to DME.^12^

As anticipated, the presence of lens opacities due to cataract significantly increased the number of imaging attempts required to get sufficient quality images, as well as the requirement for dilation, however sufficient image quality was obtained in 96.1%. While selective dilation may be a challenge to scalable clinical implementation in some cases, the operator is explicitly advised on the need for dilation by the AI system: if an operator cannot capture three images of sufficient image quality without pharmacologic dilation, the system recommends the use of dilation drops.

In this regulated pivotal trial, the AI system was compared to the highest quality reference standard as determined by the FPRC, and met predetermined sensitivity and specificity standards for the autonomous detection of more than mild DR or DME in people with diabetes but no history of DR in primary care settings.

The results, in part, led FDA to authorize IDx-DR for “for use by health care providers to automatically detect more than mild diabetic retinopathy (mtmDR) in adults (22 years of age or older) diagnosed with diabetes who have not been previously diagnosed with DR”, as, the first autonomous diagnostic AI system authorized by FDA in any field of medicine - without the need for a clinician to also interpret the image or results.^41^ At a high level, the results demonstrate the ability of autonomous AI systems to bring specialty-level diagnostics to a primary care setting, with the potential to increase access and lower cost. For people with diabetes, autonomous AI systems have the potential to improve earlier detection of DR, and thereby lessen the suffering caused by blindness and visual loss.

## Methods

### Autonomous AI diagnostic system

The autonomous AI system, IDx-DR, has two core algorithms, an Image Quality AI-based algorithm, and the Diagnostic Algorithm proper. The complete AI system was locked before the start of this study (see below).

### Image quality algorithm

The image quality algorithm is implemented as multiple independent detectors for retinal area validation as well as focus, color balance and exposure, and is used interactively by the operator to detect, in seconds, sufficient image quality for the Diagnostic algorithm to rule out (or in) mtmDR, and thus maximize the number of subjects that can be imaged succesfully. As its input it takes four retinal images, and its output is whether quality is sufficient and if not, whether this is due to field of view or image quality.^42^

### Diagnostic algorithm

The evolution of the diagnostic algorithm has been described extensively in publications spanning almost two decades.^12,18,19,43–45^ It is a clinically-inspired algorithm, and therefore has independent, validated detectors for the lesions characteristic for DR, including microaneurysms, hemorrhages and lipoprotein exudates,^40^ the outputs of which are then fused into a disease level output, using a separately trained and validated machine learning algorithm.^46^ The detectors have been implemented as multilayer convolutional neural networks (CNN),^47^ except the microaneurysm detector which is a multiscale featurebank detector,^45,47^ with substantially improved performance on a standardized laboratory dataset.^16^ In fact, in a laboratory study, its area under the receiver operator characteristics curve (AUC) of 0.980 (95% CI 0.968–0.992) was not statistically different from a perfect algorithm always outputting the truth, given the variability of the expert readers creating that truth.^46^

Each detector CNN was independently trained and validated to detect its assigned lesions from a region of a retinal image, using a total of over 1 million lesion patches from retinal images from people with and without DR.^16,48^ We consider these clinically inspired diagnostic algorithms with lesion-specific detectors for biomarkers, to be “physiologically plausible”, as they mimic the functional organization human visual cortex.^38^ Such “physiologically plausible” systems with explicit, multiple, partially dependent detectors and a separate module for the higher level clinical decision have parallels in the human and primate ventral visual cortex, with specific subregions dedicated to the detection of particular categories of objects.^50–52^ Downstream, in human experts, the higher level clinical decision is made in a part of the extrastriate cortex known as the fusiform face area, which is involved in making a clinical diagnosis from radiologic images, as has been found in functional imaging studies of radiologists when making clinical decisions.^53^

These physiologically plausible algorithms have been shown to be more robust to small perturbations in input images, possibly because they have partially dependent, and thus redundant detectors.^39^ Additionally, microaneurysms have been long recognized as the earliest retinal sign of DR that is seen on ophthalmoscopic examination, as recognized for the first time in the key paper by Friedenwald.^40^ However, decades before then, microaneurysms, and also hemorrhages, neovascularizations, IRMAs, exudates, and other abnormalities were already known to be the signs for DR.^54^ Clinicians managing DR are aware that, although the incidence and prevalence of DR vary across racial, ethnic and age categories, the above signs are constant across races and ethnicities—in other words, whether or not someone with diabetes, showing multiple retinal hemorrhages and neovascularizations is of Hispanic or non-Hispanic descent, for instance, does not affect whether the clinician will diagnose DR. Using detectors designed to detect these racially invariant biomarkers minimizes the risk of ethnic or racial bias in algorithm output.

The diagnostic algorithm uses four sufficient quality images and then takes seconds to make a clinical decision (at the point of care) and output a disease level indicating, whether more than mild DR and or macular edema is present.

#### Study design

From January 2017 to July 2017, 900 participants were prospectively enrolled in this observational study at 10 primary care practice sites throughout the United States. The study was approved by the institutional review board for each site, and all participants provided written informed consent. The study, which was funded by IDx LLC, was designed by the authors with input from the U.S. Food and Drug Administration (FDA) on the endpoints, statistical testing, and study design (see below). Emmes Corp, a contract research organization (CRO), provided overall project management, including data management and independent monitoring and auditing services for all sites. CCR, Inc., an Algorithm Integrity Provider (AIP), was contracted to lock the AI system, hold any intermediate and final results and images in escrow, and interdict access to these by the Sponsor, from prior to the start of the study until final data lock. Because the Sponsor was thus interdicted from access to the AI system, the AIP performed all necessary maintenance and servicing activities during the study as well as throughout closeout.

#### Study population

The target population was asymptomatic persons, ages of 22 and older, who had been diagnosed with diabetes and had not been previously diagnosed with DR. A diagnosis of diabetes was defined as meeting the criteria established by either the World Health Organization (WHO) or the American Diabetes Association (ADA); Hemoglobin A1c (HbA1c) ≥ 6.5% based on repeated assessments; Fasting Plasma Glucose (FPG) ≥ 126 mg/dL (7.0 mmol/L) based on repeated assessments; Oral Glucose Tolerance Test (OGTT) with two-hour plasma glucose (2-hr PG) ≥ 200 mg/dL (11.1 mmol/L) using the equivalent of an oral 75 g anhydrous glucose dose dissolved in water; or symptoms of hyperglycemia or hyperglycemic crisis with a random plasma glucose (RPG) ≥ 200 mg/dL (11.1 mmol/L).^55,56^ Exclusion criteria are listed in Table 3.

**Table 3:** Study exclusion criteria

unable to understand the study
unable to or unwilling to sign the informed consent
indicate persistent vision loss, blurred vision, or floaters
previously diagnosed with macular edema, severe non-proliferative retinopathy, proliferative retinopathy, radiation retinopathy, or retinal vein occlusion
history of laser treatment of the retina or injections into either eye, or any history of retinal surgery;
currently participating in another investigational eye study or actively receiving investigational product for DR or DME
a condition that, in the opinion of the investigator, would preclude participation in the study;
contraindicated for imaging by fundus imaging systems used in the study because of hypersensitivity to light, recently underwent photodynamic therapy, or was taking medication that causes photosensitivity

To help enroll a sufficient number of mtmDR participants for the evaluation of sensitivity, a stepwise enrichment strategy, as indicated in the prespecified protocol, was utilized mid-study to recruit sufficient numbers of mtmDR participants. The enrichment strategy sought higher risk participants with elevated HbA1c (>9.0%) levels or elevated Fasting Plasma Glucose; this enrichment was independently activated by the statistician while always remaining masked to the AI system outputs and the ETDRS disease levels. To account for any unintentional spectrum bias in the no/mild population, the study pre-defined a specificity outcome parameter to correct for any potential spectrum bias resulting from this enrichment strategy as co-primary.

#### Site initiation

All primary care sites in the study identified one or more in-house operator trainees to perform the AI system protocol (see below). After installation of the equipment by the Sponsor at the site, but before any participant was recruited, AI system operator trainees had to attest that they had not previously performed ocular imaging. Also, before start of study recruitment at each site, AI system operator trainees underwent a one-time standardized 4 h training program. They were trained how to acquire images, how to improve image quality if the AI system gave an insufficient quality output, and how to put images for analysis into the AI system. No additional training was provided to any of the AI system operators for the duration of the study. Independently, FPRC certified expert photographers were identified in geographic locations close to each site by the CRO, and documented 4W-D FPRC certification was required before any participant was imaged.^22^ The CRO independently completed site initiation visits at each site to ensure each site met all the good clinical practice requirements prior to start of enrollment.

#### Study protocol

All participants gave written informed consent to participate in both the *AI system protocol*, as well as the *FPRC imaging protocol*, using two different cameras:

The *AI system protocol* consisted of the following steps:

1. Operator takes images with a nonmydriatic retinal camera (NW400, Topcon Medical Systems, Oakland, NJ) according to a standardized imaging protocol with one disc and one fovea centered 45° image per eye;
2. Operator submits images to the AI system for automated image quality and protocol adherence evaluation;
3. If the AI system outputs *insufficient quality*, steps 1–2 are repeated until *sufficient quality* is output or 3 attempts were made. If the AI system still indicates that images are of insufficient quality, the participant’s pupils are dilated with tropicamide 1.0% eyedrops, (provided by the Sponsor at each site), until the pupil diameter is at least 5 mm in each eye or 30 minutes have passed, and steps 1–2 are repeated until *sufficient quality* is output or 3 attempts were made. If the AI system still outputs that images are of insufficient quality, the AI system output of insufficient quality is automatically provided to the CRO via secure data transfer;
4. Whenever the AI system indicates sufficient quality, the AI system disease level output (either *mtmDR detected* or *mtmDR not detected*) is automatically provided to the CRO via secure data transfer; the final AI system output provided to the CRO after this protocol was *mtmDR detected*, *mtmDR not detected* or *insufficient quality*

The FPRC imaging protocol was then conducted, and consisted of the following steps, all performed by an FPRC certified photographer:

1. If participant is not already dilated, dilating eye drops of tropicamide 1.0% are administered;
2. Digital widefield stereoscopic fundus photography is performed, using a camera capable of widefield photography, (Maestro, Topcon Medical Systems, Oakland, NJ) according to the FPRC 4W-D stereo protocol, by an FPRC certified photographer;^22^
3. Anterior segment photography for media opacity assessment is performed according to the Age Related Eye Disease Study,^57^ by an FPRC certified photographer;
4. OCT of the macula is performed using a standard OCT system capable of producing a cube scan containing at least 121 B scans, (Maestro, Topcon Medical Systems, Oakland, NJ) according to the FPRC OCT protocol, by an FPRC certified photographer.^22^

The FPRC certified photographers were masked to the AI system outputs at all times.

#### Reference standards

The FPRC grading protocol consisted of determination of ETDRS Severity Scale (SS) levels for fundus photographs and standardized OCT grading, as follows: the 4W-D images were read by three experienced and validated readers at the FPRC according to the well-established ETDRS SS, using a majority voting paradigm.^12,58^ The macular OCT images were evaluated for the presence of center-involved DME by experienced readers at the FPRC according to the DRCR grading paradigm.^24^ For each participant, the ETDRS levels were mapped to mtmDR + (ETDRS level 35 or higher and /or DME present), or mtmDR- (ETDRS level 10–20 and DME absent), taking the worst of two eyes to correspond to the outputs of the AI system at the participant level.^16^ To measure sensitivity for the cases requiring immediate followup, called vision threatening DR, we defined vtDR + as ETDRS level 53 or higher, and/or DME present, See Supplemental Table 2 for the mapping from ETDRS and DME levels to dichotomous mtmDR- and mtmDR + and vtDR +. Because DME can be identified both on the basis of retinal thickening on stereoscopic fundus photographs, as well as on the basis of retinal thickening on OCT, we separately analyzed both. Stereoscopic fundus-based Clinically Significant DME (CSDME) was identified if there was either retinal thickening or adjacent hard exudates < 600 µm from the foveal center, or a zone of retinal thickening > 1 disc area, part of which is less than 1 disc diameter from the foveal center, according to the FPRC, in any eye.^22,58,59^ OCT based center-involved DME was identified if a participant had central subfield (a 1.0 mm circle centered on the fovea) thickness that was >300 µm according to the FPRC, in any eye.^20^ Accordingly, we further specify the definition of mtmDR where relevant:

*fundus mtmDR* + is defined as

- ETDRS level ≥ 35 (determined from fundus photographs)
  and/or
- CSDME (determined from fundus photographs)

and *multimodal mtmDR* + is defined as:

- ETDRS level ≥ 35 (determined from fundus photographs), and / or
- CSDME (determined from fundus photographs) and / or
- center-involved DME (determined from OCT).

and similarly for vtDR + . FPRC readers were masked to the AI system outputs at all times, masked to the fundus photograph reading when evaluating the OCT images, and masked to OCT readings when evaluating fundus photographs.

#### Primary and secondary outcomes

The primary outcomes were the sensitivity and specificity of the AI system, which had a pre-set threshold and was locked, to detect fundus-based mtmDR + according to the FPRC grading. The CRO received all final FPRC gradings and the final AI system outputs for all participants. FPRC staff, primary care site personnel, Sponsor personnel, and the statistical team were masked at all times to the AI system outputs. There were no interim analyses. The analysis was conducted following statistical analysis plan finalization and final database lock.

#### Statistical analysis

Study success was pre-defined as both sensitivity and specificity (see below) of the AI system in the US diabetes population. The hypotheses of interest were

$$H_0\,:\,p\, < \,p_0\,{\mathrm{vs}}{\mathrm{.}}\,H_A\,:\,p\, \ge \,p_0$$

where *p* is the sensitivity or specificity of the AI system and *p*_0_ = 75% for the sensitivity endpoint and *p*_0_ = 77.5% for the specificity endpoint under the null hypotheses. The alternative hypotheses were 85% for sensitivity and 82.5% for specificity, reflecting anticipated enrollment numbers, and pre-specified regulatory requirements. One-sided testing was further pre-specified for both sensitivity and specificity; a one-sided 2.5% Type I error was used resulting in a one-sided 97.5% rejection rule per hypothesis. To preserve Type I error, study success was defined as requiring both null hypotheses to be rejected at the end of the study, e.g.

$$P_\pi \left( {H_A\left| {{\mathrm{Data}}} \right.} \right) > 0.975.$$

The primary sensitivity calculation was performed using a logistic regression model including all mtmDR participants without any baseline covariate adjustment while the primary specificity calculation was performed using a logistic regression model with enrichment as a baseline covariate. A Firth adjustment was used to project sensitivity without any baseline covariate adjustment while the specificity was projected using absent enrichment status to diffuse spectrum bias^60^; enrichment was intended to increase the number of mtmDR cases based on stepwise increase of HbA1C levels, and thus expected to cause enrichment spectrum bias. Therefore, the specificity calculation was prespecified to correct for such spectrum bias; no such correction was prespecified for sensitivity analysis, because the goal was to shift the frequency of more severe DR cases. No data imputation was used for primary analyses.

Analyses were based on the data from the ITS population: participants who had valid results on both the FPRC imaging and reading protocol, and the AI system output, except where indicated; reported subgroup analyses were prespecified; subgroups < 10 participants are not reported. Results are reported as posterior means, medians and with corresponding two-sided 95% confidence intervals (CI). All analyses were conducted with the use of SAS software, version 9.1. Sample sizes for these hypotheses were calculated for at least 85% power and one-sided 2.5% Type 1 error, requiring samples of 149 mtmDR positive participants and 682 mtmDR negative DR participants.

The study protocol and statistical analysis plan are available in the Supplementary information.

### Code availability

The AI system described in this study is available as IDx-DR from IDx, LLC, Coralville, Iowa. The underlying source codes are copyrighted by IDx, LLC, and are not available. No other custom code was used in the study.

### Data and materials availability

The datasets generated during the current study that were used to calculate the primary outcome parameters are available upon reasonable request from the corresponding author, M.D.A., as well as from P.T.L.

## Electronic supplementary material

IDx-DR pivotal study protocol

IDx-DR pivotal study SAP Supplemental Tables 1 and 2

Supplemental Tables 1 and 2

## Electronic supplementary material

**Supplementary information** accompanies the paper on the npj Digital Medicine website (10.1038/s41746-018-0040-6).
