# Supplementary material for: Pivotal trial of an autonomous AI-based diagnostic system for detection of diabetic retinopathy in primary care offices
Source: NPJ Digit Med. 2018 Aug 28;1:39. doi: 10.1038/s41746-018-0040-6 (PMC6550188; doi:10.1038/s41746-018-0040-6)
Supplement: Supplementary file 1 — IDx-DR pivotal study protocol [file 41746_2018_40_MOESM1_ESM.pdf]

STATISTICAL ANALYSIS PLAN SYNOPSIS

August 20, 2017

A MULTICENTER STUDY TO EVALUATE PERFORMANCE OF AN AUTOMATED DEVICE FOR THE DETECTION OF DIABETIC RETINOPATHY

Version 2 (Synopsis)

Philip Lavin PhD, FASA, FRAPS

For

IDx LLC
485 Highway 1 W
Iowa City IA 52246

CONFIDENTIALITY STATEMENT

This document contains information that is the property of IDx and therefore is provided to you in confidence for review by you, your staff, and regulatory authorities. It is understood that this information will not be disclosed to others without written approval from IDx.

1. BACKGROUND

The IDx-DR device is intended to be used by health care providers to automatically screen for more than mild diabetic retinopathy (mtmDR). The IDx-DR outputs correlate with levels of disease identified in the America Academy of Ophthalmology's Preferred Practice Pattern (AAO PPP), which are associated with well-characterized management recommendations [1]. According to the AAO PPP, patients presenting with mtmDR have a level of disease associated with management recommendations outside of the normal 12-month screening period for patients with diabetes, and the physician overseeing the exam is advised to refer them to an eye care provider.

The IDx-DR Device, will produce a report for the physician overseeing the exam. IDx-DR will produce **one** of three outputs:

1. The IDx-DR result MORE THAN MILD DIABETIC RETINOPATHY DETECTED: REFER TO AN EYE CARE PROFESSIONAL indicates the presence of more than mild diabetic retinopathy, which includes moderate non-proliferative diabetic retinopathy, severe non-proliferative retinopathy (NPDR), proliferative retinopathy (PDR), and/or macular edema. Per the AAO PPP, patients with mtmDR have a level of disease associated with management recommendations outside of the normal 12-month screening period for patients with diabetes, and the physician overseeing the exam is advised to *refer* them to an eye care provider.

2. The IDx-DR result of NEGATIVE FOR MORE THAN MILD DIABETIC RETINOPATHY: RESCREEN IN 12 MONTHS will indicate that IDx-DR did not detect macular edema, moderate non-proliferative retinopathy, severe non-proliferative retinopathy, or proliferative retinopathy. Per the AAO PPP, it is recommended that patients be *rescreened annually*.

3. The IDx-DR report will indicate EXAM QUALITY INSUFFICIENT when the IDx-DR Exam Quality Index determines that the result should not be provided to the user due to insufficient fundus image quality submitted for analysis. The Exam Quality Index threshold was designed to determine when disease cannot be effectively ruled out from a set of exam images. The device will give the user guidance on what images caused the problem so the user can take action to efficiently re-image the patient either non-mydriatically or in conjunction with the use of pharmacologic dilation. Guidance to the user will be expressed as "Insufficient Quality" (an indication of dark or obscured images) or "Protocol Failure" (an indication that the submitted images do not adhere to the protocol of one fovea centered and one macula centered image per eye). Patients who are unable to receive a result other than "EXAM QUALITY INSUFFICIENT" after reimaging will ultimately be recommended for *referral* to an eye care professional in product labeling.

In rare cases, the IDx-DR Device may also produce a result of "Technical Failure – Exam not Suitable for Analysis." This occurs when the exam submitted is of such poor quality that IDx-DR is unable to recognized submitted images as fundus photographs and therefore cannot analyze the image due to insufficient quality. Note that this result is different from "Exam Quality Insufficient," which is a result where the IDx-DR exam quality index is designed to override a diagnostic result that would otherwise be produced.

The IDx goal is to deliver a level of performance that aligns with the FDA's target that the IDx-DR Device rule out lower bounds of at least 75% sensitivity and at least 77.5% specificity for the mtmDR configuration, with study success defined as achieving at least 80% sensitivity and at least 80% specificity.

All diagnoses will be made according to the Fundus Photograph Reading Center (FPRC).

2. PROTOCOL OVERVIEW

2.1. Study Design

2.1.1. Prospective Cohort

This is a multi-center pivotal study for registration purposes. Study subjects (n=845 after exclusions) inclusive of 189 mtmDR subjects and 656 no/mild subjects) will be recruited at primary care sites. Between 10 and 15 independent primary care sites will participate in the study.

2.1.2. Enriched Cohort

At the start of the study all participants with diabetes who meet inclusion and exclusion criteria will be enrolled. To avoid excessive enrollment in any one stratum (no/mild, mDR, or vtDR), the totals will be monitored monthly and the study population may be enriched to ensure sufficient numbers of subjects with mtmDR. Patient enrichment will be targeted based on elevated HbA1C or fasting blood glucose (FBG), factors which have been shown to be correlated with higher rates of vtDR among patients with diabetes. No cap will be placed on enriched patients since such patients will be prospectively recruited.

The impact of enrichment based on elevated HbA1C is not known. The goal will be to recruit enough subjects to satisfy each stratum. If enrollment patterns are not on track with the study's target population after monthly reviews, enrollment at all sites will be narrowed to participants with an HbA1c of 9% or higher. Adjustments from this level may be instituted after monthly review of the number of participants with diabetic retinopathy or diabetic macular edema. Study sites will be notified by the CRO the first work day of each month if there is to be a change in the HbA1c threshold.

Monitoring of strata will be performed by the statistician while maintaining masking to IDx-DR results; only HbA1C, baseline covariates, and FPRC categorizations will be visible for each subject. The statistician (team) will notify the CRO before the first of each month whether an update HbA1C threshold is needed. In the event that the strata targets are reached, then the HbA1C threshold will be turned off.

2.1.3. Precision Study

A repeatability and reproducibility "sub-study" will be defined in a separate protocol and SAP.

2.2. Independent Reading Center

The FPRC independent Reading Center will determine the reference standard for the severity of retinopathy and diabetic macular edema according to the Early Treatment of Diabetic Retinopathy Grading System (ETDRS) based on 4-W fundus photographs. Center involved macular edema will be assessed from OCT images according to the RC protocol, to be used for secondary endpoint analysis. Incidental findings and an indication of media opacity presence will also be documented.

2.3. Statistical Monitoring

The statistician will compare the performance of the IDx-DR device to FPRC readings in terms of sensitivity and specificity as well as Receiver Operator Characteristics for the device's mtmDR classifier algorithm. Results will also be monitored for enrichment outcomes.

3. STATISTICAL ASSUMPTIONS AND SAMPLE SIZE CALCULATIONS

3.1. Classifier Algorithms

There are separate internal classifier algorithms for:

- Algorithm A (vtDR output): differentiating vtDR vs. combined mDR + mild DR + no DR, and
- Algorithm B (mtmDR output): differentiating mtmDR vs. combined mild DR + no DR.

These internal algorithms can be separately validated, though only the mtmDR configuration will be studied for this protocol and SAP.

Per the FPRC's Grading Charter, the following table summarizes the conversion of ETDRS scores to ground truth for the study:

IDx-DR Output	AAO PPP	ETDRS DR Grades	ETDRS Macular Edema Determination

No/Mild DR		Normal or Minimal NPDR	10, 12, 14A, 14B, 14C, 14Z, 15, 20	
		Mild NPDR		
mtmDR		Moderate NPDR	35A, 35B, 35C, 35D, 35E, 35F, 43A, 43B, 47A, 47B, 47C, 47D	Definite Criteria 1: zone of retinal thickening > 1DA, part < 1 DD from center
	vtDR	Severe NPDR	53A, 53B, 53C, 53D, 53E, 60, 61A, 61B, 65A, 65B, 65C, 71A, 71B, 71C, 71D, 75, 81, 85A, 85B	Definite Criteria 2: retinal thickening or adjacent HE < 600μ from center
		Non-high-risk PDR		
		High Risk PDR		
		Macular Edema		

Note that subjects with a grade of 90 will be excluded from primary analysis as being "ungradable" by FPRC for diabetic retinopathy severity level.

Note that all analyses will also be re-run with ETDRS 12s excluded from analysis.

3.2. Co-primary Effectiveness Endpoints

Sensitivity and specificity will be the co-primary effectiveness endpoints to be evaluated against FPRC determination (truth) in the ITS (Intent to Screen) population for whom IDx-DR provides a diagnostic result (primary) against pre-established standards (null hypotheses) to be rejected if too low relative to these pre-defined thresholds.

The computations for the sensitivity and specificity measures are displayed in Table 4.7.1. An mtmDR subject with a positive algorithm counts towards the sensitivity numerator while a Mild + No DR subject with a negative algorithm will count towards the specificity numerator. Every case will be accounted for in the sensitivity and specificity calculations.

3.3. Closed Testing Strategy

The closed testing strategy requires statistical significance to be separately met for both sensitivity and specificity.

3.4. IDx Algorithms

The IDx-DR Device and its algorithm will be locked prior to study launch. A version of the tested software will be placed in third-party escrow during the study, and will be available for comparison to the algorithm used in the study if questions of algorithm alterations arise. IDx-DR will receive and process all fundus images in accordance with the clinical trial imaging procedure. The test results will include: mtmDR positive and mtmDR negative, insufficient exam quality – and, in rare cases, a technical failure.

3.5. Enrichment Strategy

Prospective enrichment will be used. Any adjustment of the HbA1c threshold will be noted and the comparability of baseline characteristics corresponding to any change will be presented for ethnicity and race, cataract, incidental findings, and mtmDR characteristics. Comparability will be assessed using two-sided 95% confidence intervals for the respective cohorts (enriched vs. not enriched).

3.6. Key Assumptions

The following assumptions govern sample size:

- There will be a separate algorithm for mtmDR with complementary indication for use. All subjects will be included in analyses for either sensitivity or specificity.
- The algorithms will be locked prior to subject enrollment.
- Prospective enrichment will be allowed on the basis of elevated A1c or elevated FBG levels.
- The primary population for analysis will be patients from the Intention to Screen (ITS) population that have an IDx-DR result and a reference standard from the Reading Center.
- It is estimated that less than 10% of subjects will be unimageable in a PCP setting, meaning more than 90% of subjects imaged in the ITS population will have a diagnostic result from the IDx-DR Device.
- To address incomplete data, prospective rules for case inclusion are summarized (see Section 4.7.7.2)

  Both sensitivity and both specificity must achieve statistical significance to support study success. This strategy preserves the overall Type I error.

The statistical assumptions that were used to plan the study are as follows:

- Sample size calculations were performed at the subject-level.
- Use of conservative one-sided hypothesis testing with overall one-sided 2.5% Type I error and 85-90% power to rule out the targeted 77.5% specificity lower bound for combined mild DR + no DR and the targeted 75% sensitivity lower bound for mtmDR.
- The null hypotheses will be tested separately for mtmDR sensitivity and mtmDR specificity.
- All combined mild DR + no DR subjects will be included in the mtmDR specificity calculation. All mtmDR (inclusive of vtDR) subjects will be included in the more than mild DR sensitivity calculation.
- Subjects that received a IDx-DR result of “Insufficient Exam quality” will be excluded from primary analysis. We will determine their characteristics in the secondary ITS calculations: one where these results are considered under the “best case scenario,” where all missing IDx-DR results, including insufficient exam quality and technical failures, completely agree with the FPRC reference results and one where they are considered “worst case scenario,” where all missing IDx-DR results, including insufficient exam quality and technical failures, completely disagree with the RC reference results. As with all subjects, these subjects will also have been imaged using a 4-W protocol, with the wide field photos used by the FPRC to determine the reference standard.

3.7. Hypothesis Tests

The null hypotheses to be tested against a higher alternative hypothesis for the ITS population follow:

- mtmDR sensitivity: ≤75% null hypothesis vs >75% alternative hypothesis (85% used for sample size calculation)
- mtmDR specificity: ≤77.5% null hypothesis vs >77.5% alternative hypothesis (82.5% used for sample size calculation)

3.8. Pivotal Study Sample Size Calculations

The following sample sizes for mtmDR sensitivity and combined mild DR + no DR specificity are summarized below in Table 3 where one-sided 2.5% Type 1 error and a minimum of 85% power have been assumed. The total sample size of 845 evaluated subjects includes 189 mtmDR subjects and 656 combined mild DR + no DR subjects after losses.

As losses are confirmed, subjects will be replaced. Final sample sizes including hypothetical 10% losses (210 and 729) for a total of 939 subjects are shown below. This sample size includes enrollment from enrichment. The loss rate for FPRC losses and IDx algorithm insufficiencies will be monitored in a masked manner to protect against over-enrollment.

Exact binomial test: One-sided 2.5% alpha and 85-90% power

	**Sensitivity**	**Specificity**
**Null hypothesis proportion,** $\square_0$	0.750	0.775

**Alternative proportion, $\square_A$**	0.850	0.825
**Power (%)**	85	90
**N (after losses)**	189	656
**N (before 10% losses)**	210	729

Corresponding statistical significance (one-sided p=0.025) will be achieved as displayed below.

Exact binomial test: Reaching one-sided p=0.025

	Sensitivity	Specificity
**Null hypothesis proportion, $\square_0$**	0.750	0.775
**Success proportion, $\square_A$**	0.812	0.807

4. STATISTICAL METHODS

This analysis plan describes the planned data analyses.

4.1. General Considerations

- This Statistical Analysis Plan (SAP) is based on the Clinical Trial Protocol for **A MULTICENTER STUDY TO EVALUATE PERFORMANCE OF AN AUTOMATED DEVICE FOR THE DETECTION OF DIABETIC RETINOPATHY AND DIABETIC MACULAR EDEMA** dated May 2017.
- All calculations will be performed using SAS statistical software, version 9.1 or later, and StatXact, version 10 or later.
- Final analysis will be conducted after the last subject completes evaluation by the RC.
- Analyses will be conducted at the subject-level (since both eyes are required to run the IDx algorithm).
- Study data will be used to simultaneously evaluate the mtmDR algorithm with study success defined to be rejecting both study hypothesis (see Section 4.7).
- All hypothesis tests will account for all subjects as previously stated (see Section 3.6).
- Testing will be performed for the ITS population for whom IDx-DR delivers a screening result and full ITS (secondary) to support labeling.
- To be included in ITS effectiveness analyses, subjects must be declared evaluable according to the FPRC and have a valid IDx-DR test.
- To be included in the primary effectiveness analyses, subjects must be declared evaluable according to the FPRC and have an IDx-DR screening test result.
- One-sided 2.5% Type 1 error was used to plan sample size.
- One-sided p=0.025 will be required to achieve statistical significance for effectiveness; no p-value adjustment will be made for testing effectiveness endpoints since both specificity and sensitivity criteria must be met for study success.
- There are 18 effectiveness endpoints of interest (see Section 4.7.4) with two primary and six secondary endpoints.
- The literature suggests that there will be five times as many no DR cases as mild DR cases.
- The effect of subject losses will be assessed using robustness analyses for the two primary effectiveness endpoints (specificity and sensitivity).
- Verification bias will be investigated in excluded subjects.
- All effectiveness analyses will be performed using the ITS population for who IDx-DR delivers a screening result. Additional analyses will support labeling claims in the absence of a diagnostic result due to low quality imaging in a PCP setting by previously untrained ophthalmic photographers.
- Enriched subjects will be analyzed in combination with other subjects as well as separately to assess their impact.

4.2. Study Objectives

The study objectives are to establish mtmDR specificity and the corresponding sensitivity.

4.3. Photo Acquisition

The study population is described at the subject level. All subjects will be evaluated by photography and these photos will be subsequently evaluated via IDx-DR so no formal safety events are expected. Subjects may be reimaged per the IDx-DR imaging procedure if the initial image quality is not sufficient and the subject is willing to be retested.

4.4. Analysis Populations

The following database components will be used to determine effectiveness analysis population inclusion:

- Subject eligibility
- IDx-DR results
- FPRC evaluation.

Each data source will be reviewed by the Contract Research Organization (CRO) for the algorithm processing and results reflecting input from appropriate medical, clinical, data management, and statistical personnel. Subject inclusion / exclusion status with respect to the study populations will be made without knowledge of IDx-DR outcome.

4.4.1. Intent-to-screen (ITS) Population

The ITS population is described at the subject level and represents all eligible subjects with a Reading Center reference standard result. Study eligibility will be ascertained to exclude ineligible subjects from the ITS population. The primary study analyses will include the ITS population for whom IDx-DR delivers a screening output (i.e., a result of mtmDR or no/mild DR). Additional ITS population analyses will be confirmatory relative to these primary analyses for assessment of effectiveness, with reference standard established by the FPRC, which will determine the DR category each subject belongs to: vtDR, mDR, mild DR, and no DR, even if IDx-DR cannot provide a result due to Insufficient Exam Quality. Data for these calculations will be imputed per Section 4.7.7.2, with cases being considered referrals (i.e., TPs and FPs) or missed cases (i.e., FNs and TNs) as well as cases being considered under the best and worst case scenario, in terms of agreement with the reference standard.

Rules regarding ITS exclusion decisions will be prospectively established prior to database lock and reviewed by individuals without knowledge of the IDx-DR outcome by subject.

4.5. Subject Disposition

All enrolled subjects will be included in summaries of disposition and evaluation for analysis sets. Accounting will be provided for the following parameters:

1. Number of subjects by study site including those that receive a result of insufficient exam quality.
2. Numbers of subjects according to eligibility status overall and by study site.
3. Number of subjects able to be evaluated for diagnostic category (including enriched subjects).
4. Number of subjects with useable photos for the IDx-DR evaluation.
5. Construction of the ITS populations by subject from exclusions.
6. Study completion status and reasons for discontinuation by subject.

Subject exclusions from analysis sets and associated reasons will be tabulated.

The protocol deviations leading to ITS exclusions will be listed.

4.6. Demographic and Other Baseline Characteristics

Sex, age, race, and ethnicity will be presented using descriptive statistics by sites and by category for the overall ITS population and for the respective cohorts. Baseline characteristics will be compared first within the prospective enrichment cohort; any adjustment of the HbA1c threshold will be noted and the comparability of baseline characteristics corresponding to any change will be presented for ethnicity and race, cataract, incidental findings, and mtmDR characteristics. Cohort comparability will be assessed across cohorts using two-sided 95% confidence intervals for the paired differences between cohorts; a logistic regression model will be used for binary baseline measures as the dependent outcome, while an ANOVA model will be used for continuous measures as the dependent outcome.

4.7. Effectiveness Analyses

4.7.1. Statistical Terminology

The measures of screening accuracy are defined as follows:

		Reference Standard Panel	
	Outcome	Positive	Negative
Test	Positive	TP	FP
	Negative	FN	TN

- Sensitivity = TP/(TP+FN)
- Specificity = TN/(TN+FP)
- Positive predictive value (PPV) = TP/(TP+FP)
- Negative predictive value (NPV) = TN/(FN+TN)
- Positive likelihood ratio (PLR) = [TP/(TP+FN)]/[FP/(FP+TN)]
- Negative likelihood ratio (NLR) = [FN/(TP+FN)]/[TN/(FP+TN)]
- Odds ratio (OR) = (TP/FP)/(FN/TN)

4.7.2. Effectiveness Analysis Strategy

A frequentist analysis approach will be used. One-sided 97.5% confidence intervals will be computed. P-values will be computed.

Analysis will be run in SAS in a GCP controlled environment.

4.7.3. Final Analysis

The generalized hypotheses of interest are

$$H_0: p \leq p_0 \text{ vs. } H_A : p > p_0$$

where p is the sensitivity or specificity of the proposed diagnostic test and $p_0 = 75\%$ for the sensitivity endpoint and $p_0 = 77.5\%$ for the specificity endpoint under the respective null hypothesis. One-sided testing will be performed for both sensitivity and specificity; one-sided 2.5% Type I error will be used which results in a one-sided 97.5% rejection rule per hypothesis.

4.7.4. Primary Effectiveness Endpoints

The primary effectiveness endpoints for this study are the sensitivity and specificity. One-sided 97.5% confidence intervals for the lower bounds will be calculated for these binary measures. Descriptive statistics will be presented for the percent positive separately for each of mtmDR and no/mild DR. Results will be displayed per cohort and combined. All subjects from both cohorts will be placed into and analyzed according to the respective vtDR, mDR, mild DR, and no DR classifications established according to the FPRC grading charter.

In calculating the mtmDR sensitivity and specificity, the estimation analyses will be carried out on the combined cohorts. The impact of the following baseline covariates will be considered to evaluate cohort effect using a logistic regression model: age (<55 years vs $\geq$55 years), gender, race, ethnicity, BMI (<30 kg/m^2 vs $\geq$30 kg/m^2, if available), HbA1c (<10% vs $\geq$10%), diabetic

interval since diagnosis (<10 years vs ≥10 years, if available), and cohort (not enriched, enriched). Results will be reported as posterior means, medians and with corresponding one-sided 97.5% confidence intervals (CI).

Analyses will be run for the ITS population with and without imputation for "Insufficient Exam Quality" cases. The ITS population analysis will be primary based on subjects with an IDx-DR screening result, with secondary analyses preforming different imputations for those patients with a result of "Insufficient Exam Quality".

The following additional analyses will be performed separately for sensitivity and specificity per cohort and pooled with a single covariate included for the enriched cohort in the respective logistic regression models.

4.7.5. Secondary Effectiveness Endpoints

Additional, non-primary analyses will also be performed to assess the following overall and by cohort:

- The predictive value of a positive IDx test for mtmDR
- The predictive value of a negative IDx test for mtmDR:
- The positive and negative likelihood ratios for mtmDR.

Analysis will also be performed considering the "best case" and "worst case" scenario for missing data, specifically where there is no IDx-DR disease output or FPRC has graded the exam an ETDRS 90. Analysis will also be performed excluding exams graded by the FPRC as an ETDRS 12 - the special grade designation reserved by the FPRC for cases where "Non-DR Abnormalities" are detected in the absence of DR by readers. See below for endpoint calculations.

4.7.6. Effectiveness Analysis Methodology Summary

There will be two primary, six secondary, and 10 confirmatory effectiveness endpoints as summarized below. The p-values will be computed for all subjects combined for the ITS population. The following parameters and associated hypotheses will be used to assess IDx effectiveness:

Parameter	Hypothesis/ Analysis*
Endpoint 1 (Primary) and 2 (Confirmatory): Specificity, evaluated for combined mild DR + no DR (mtmDR specificity). ITS with IDx-DR result is primary and with imputations for "Insufficient Exam Quality" confirmatory.	The null hypothesis for specificity is 77.5% for combined mild DR + no DR (mtmDR specificity). A one-sided 97.5% lower confidence bound from an exact binomial model will be computed for the specificity.
Endpoints 3 (Primary) and 4 (Confirmatory): Sensitivity, separately evaluated for mtmDR. ITS with IDx-DR result is primary and with imputations for "Insufficient Exam Quality" and the special case of ETDRS 12s as confirmatory.	The null hypothesis for sensitivity is 75% for mtmDR. A one-sided 97.5% lower confidence bound from an exact binomial model will be computed for the sensitivity. Results will be evaluated with and without the enriched population.
Endpoints 5-6 (Secondary) and 7-8 (Confirmatory): The predictive values of a positive IDx test (PPV), and a negative IDx test (NPV). ITS with IDx-DR result is secondary and with imputations for "Insufficient Exam Quality" and the special case of ETDRS 12s as confirmatory.	One-sided 97.5% confidence intervals for each predictive value will be computed using an exact binomial distribution. Results will be evaluated with and without the enriched population.
Endpoints 9-10 (Secondary) and 11-12 (Confirmatory): The positive (PLR) and negative (NLR) likelihood ratios of IDx-DR. ITS with IDx-DR result is secondary and with imputations for "Insufficient Exam Quality" and the special case	One-sided 97.5% confidence intervals for each measure will be computed using StatXact. Results will be evaluated with and without the enriched population.

of ETDRS 12s as confirmatory.	
Endpoints 13-14 (Confirmatory): Primary endpoints will be calculated again without exams graded ETDRS 12 by FPRC.	The null hypothesis for specificity is 77.5% for combined mild DR + no DR (mtmDR specificity). The null hypothesis for sensitivity is 75%.

*Hypothesis tests will be limited to the primary effectiveness endpoints.

4.7.7. Further Effectiveness Analyses

4.7.7.1. Ineligibility Impact

There will be a detailed accounting of every subject to assess the impact of ITS exclusions. The nature of the loss being missing at random will be evaluated. Age, sex, race, and ethnicity will be assessed for any impact on exclusions due to ineligibility, as will an analysis based on incidental findings indicated by the Reading Center from either fundus photos or anterior segment images. These analyses focus on sensitivity and specificity.

4.7.7.2. Missing Data Handling

The primary analysis for ITS and the secondary analysis will be based on all available data without imputation for missing data. Results will be calculated for the mtmDR configuration of the product. The following table illustrates the different ways that missing results will be calculated as confirmatory analyses.

	**IDx-DR Screening Result was Obtained**	**IDx-DR Obtained a Result of "Insufficient Exam Quality"**
**FPRC Truth was Obtained**	No missing results – see primary analysis	1) IDx-DR "Insufficient Exam Quality" results will be counted as being in full agreement with the reference standard<br>2) IDx-DR "Insufficient Exam Quality" results will be counted as being in full disagreement with the reference standard<br>3) IDx-DR "Insufficient Exam Quality" results will be counted as being positive for disease<br>4) IDx-DR "Insufficient Exam Quality" results will be counted as being negative for disease<br>5) IDx-DR result provided as if Exam Quality was turned off will be compared to FPRC truth (note, indices will not be provided for image failure)
**FPRC Truth was Not Obtained**	1) IDx-DR compared to FPRC and missing FPRC truth assigned IDx-DR result<br>2) IDx-DR compared to FPRC and missing FPRC truth assigned opposite of IDx-DR result<br>3) IDx-DR result compared to FPRC and missing FPRC truth counted as all being normal<br>4) IDx-DR result compared to FPRC and missing FPRC truth counted as all being diseased	1) IDx-DR compared to FPRC and missing FPRC truth counted as all being normal, missing IDx-DR result counted as being in full agreement with FPRC<br>2) IDx-DR compared to FPRC and missing FPRC truth counted as all being diseased, missing IDx-DR result counted as being in full agreement with FPRC<br>3) IDx-DR compared to FPRC and missing FPRC truth counted as all being normal, missing IDx-DR result counted as being in full

		disagreement with FPRC<br>4) IDx-DR compared to FPRC and missing FPRC truth counted as all being diseased, missing IDx-DR result counted as being in full disagreement with FPRC<br>5) Use IDx-DR result provided as if Exam Quality was turned off and missing FPRC counted as normal (note, indices will not be provided for image failure)<br>6) Use IDx-DR result provided as if Exam Quality was turned off and missing FPRC counted as disease (note, indices will not be provided for image failure)

Note that all analyses will also be re-run with ETDRS 12s excluded from analysis.

Further analyses will be performed to evaluate the impact of missing diagnostic data as well as missing IDx-DR outcomes by comparing demographic data such as age, sex, race, and ethnicity. Enrolled subjects will be evaluated for bias by examination of cross-tabulations of baseline demographics for IDx-DR test outcome (present vs. absent) and ITS population with and without imputation for results of "Insufficient Exam Quality" will be displayed to assess bias. In addition, the impact of population exclusions will be assessed for both sensitivity and specificity. The impact will be assessed overall and separate analyses per cohort.

4.7.7.3. Study Completion

Subjects who complete their study-directed photos in primary care and who complete gold standard ophthalmic imaging will be considered complete. The date of study completion is the date of the completed 4-W imaging.

Subjects who do not undergo their IDx-DR evaluation in primary care evaluation, and/or do not present 4-W photos that can be evaluated by the Reading Center will be discontinued from the study. The reason for the discontinuation will be recorded. The study will end when all subjects have completed the study-directed evaluations or have discontinued including withdrawals.

4.7.7.4. Verification Bias

Verification bias is a type of measurement bias in which the results of a diagnostic test affect whether the gold standard procedure is used to verify the test result. This type of bias is also known as "work-up bias" or "referral bias". In clinical practice, verification bias is more likely to occur when a preliminary diagnostic test is negative. Here, the IDx test is not invasive nor does it carry a higher risk. Verification bias is minimized in this setting since all subjects will have a gold standard test. Thus neither sensitivity nor specificity estimates may be biased. Excluded subjects will be compared to included subjects as part of this bias assessment. The impact will be assessed overall and separate analyses per cohort.

4.7.7.5. Spectrum Bias

Spectrum bias will be investigated for enriched vs. enrolled mtmDR as well as across study sites because each site may have a different case mix. This is a form of a sampling bias. Subgroup sensitivities and specificity will also be investigated. In addition, the positive and negative likelihood ratios will also be checked. Spectrum bias has two potential causes:

1. A change in the case-mix of those mtmDR subjects which may affect sensitivity.
2. A change in the case-mix of the no/mild DR subjects which may affect specificity.
3. A change in the case-mix of those without the target disorder (disease-free) may affect specificity.

Spectrum bias will be investigated for each cohort as well as across study sites because each cohort or site may have a different case mix. This is a form of a sampling bias. Subgroup sensitivities and specificities will also be investigated. In addition, the positive and negative likelihood ratios will also be checked.

4.7.7.6. Handling of Withdrawals

Subjects may be withdrawn from the study by any of the following mechanisms:

1. Voluntary withdrawal of consent to participate by the subject at any time during the study.
2. Determination by the clinical investigator that it is in the best interest of the subject.
3. Determination by the sponsor or CRO it is in the best interest of the subject.
4. Subject ineligibility.
5. Inability for the subject to come in for 4-W photos.

All data available from enrollment to subject withdrawal will be collected unless the subject withdraws consent from the study. Data will be collected for subjects who discontinue participation but who do not withdraw consent.

Bias will be assessed by looking at baseline characteristics for withdrawals due to inability of the Reading Center to perform their evaluation. Withdrawal rates will be compared per cohort, pooled, and per FPRC diagnosis.

4.7.7.7. Multiple Comparisons / Multiplicity

No p-value adjustments will be performed for multiple testing. P-values will be computed for the primary and secondary effectiveness endpoints. The other effectiveness endpoints will be displayed with distributions or one-sided 97.5% confidence intervals.

4.7.7.8. Examination of Subgroups

Results for the two primary effectiveness endpoints will be displayed by sex, age, race, and ethnicity using descriptive statistics. Enriched cases will similarly be compared to prospectively enrolled subjects.

4.7.7.9. Performance Data Stratified by Media Opacities Grading

Results will include the grading of media opacities and performance data stratified by the media opacity grading provided by FPRC.

5. CHANGES IN THE CONDUCT OF THE STUDY OR PLANNED ANALYSES

The study has dropped the vtDR algorithm due to insufficient numbers of vtDR cases that is expected to be enrolled by completion of the planned recruitment.

6. REFERENCES

1. SAS Version 9 or later, Cary NC.

2. StatXact Version 10 or later, Cambridge MA.

3. Abramoff, M. D., J. C. Folk, D. P. Han, J. D. Walker, D. F. Williams, S. R. Russell, P. Massin, B. Cochener, P. Gain, L. Tang, M. Lamard, D. C. Moga, G. Quellec and M. Niemeijer (2013). "Automated analysis of retinal images for detection of referable diabetic retinopathy." JAMA Ophthalmol 131(3): 351-357.

Proposed Analysis IDx-DR mtmDR Output

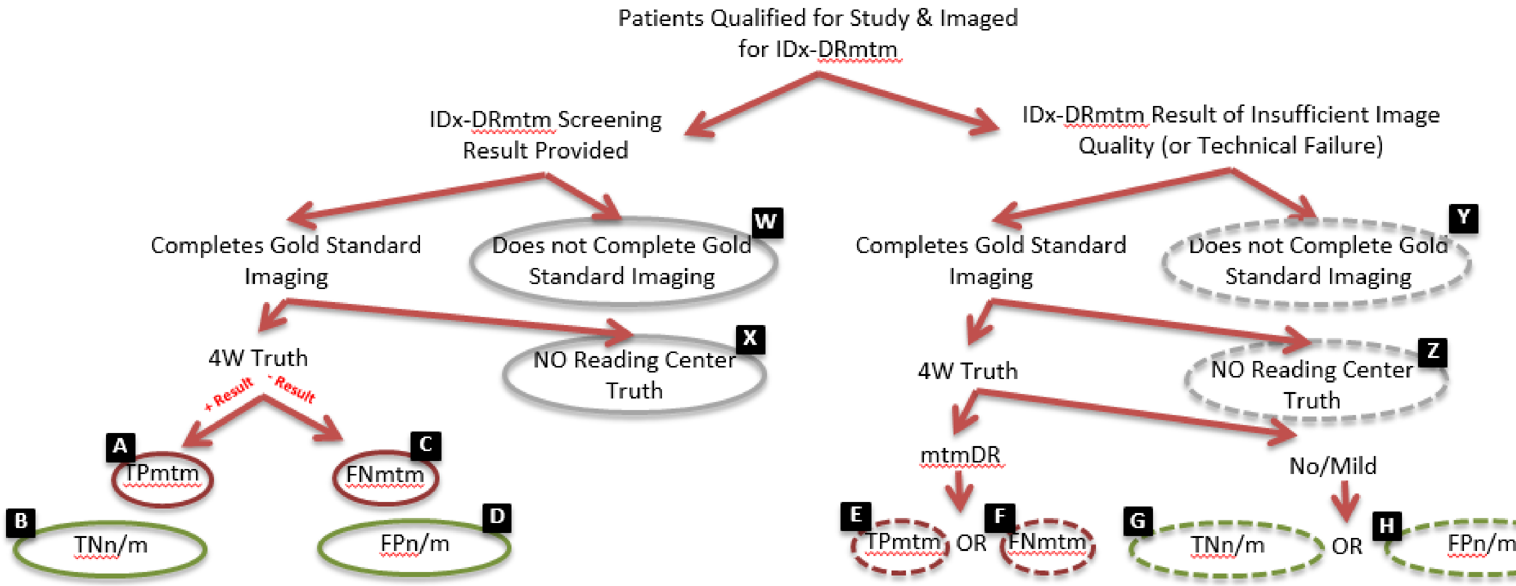

PRIMARY ENDPOINT CALCULATIONS

A /(**A** + **C**) = mtmSens
D /(**B** + **D**) = mtmSpec

Additional analysis for cases without Reading Center Truth

Best Case(s)
TP = **A** + **W** + **X**
TN = **D** + **W** + **X**

Worst Case(s)
FN = **C** + **W** + **X**
FP = **B** + **W** + **X**

SECONDARY ENDPOINT CALCULATIONS

Insufficient Image Quality treated as mtmDR+ (Appropriate Referrals)	Insufficient Image Quality treated as No/Mild (Missed Cases)	Insufficient Image Quality treated as 100% Agreement w/ Standard (Best Case)	Insufficient Image Quality treated as 100% Disagreeme with Standard (Worst Case
mtmSpec = (**A** + **E**)/(**A** + **C** + **E**)	mtmSpec = **A** / (**A** + **C** + **F**)	mtmSpec = (**A** + **E**)/(**A** + **C** + **E**)	mtmSpec = **A** / (**A** + **C** + **F**)
mtmSpec = **D** / (**D** + **B** + **H**)	mtmSpec = (**D** + **G**)/(**B** + **D** + **G**)	mtmSpec = (**D** + **G**)/(**B** + **D** + **G**)	mtmSpec = **D** / (**D** + **B** + **H**)

Additional analysis for cases without Reading Center Truth

Best Case(s)
TP = **A** + **E** + **W** + **X** + **Y** + **Z**
TN = **A** + **E** + **W** + **X** + **Y** + **Z**

Worst Case(s)
TP = **D** + **G** + **W** + **X** + **Y** + **Z**
TN = **B** + **H** + **W** + **X** + **Y** + **Z**

Appendix 1: SAP Supplement, November 10, 2017

ENROLLMENT

Enrollment details are captured from provided raw datasets ecidxb and ecidxz.
If raw.ecidxb.ENRENROD=1 or raw.ecidxz.ENRENROD=1 then Enrollment=”Yes”.

1. ITS POPULATION

ITS Population includes all enrolled subjects with an FPRC Result.

2. ENRICHMENT

Enrichment is derived on basis of an algorithm that utilizes the Start Date for the subject and the HbA1c values.

- If subject start date is between Beginning of study and February 14th @ 8:00am then Enrichment=”Not Enriched”.
- If subject start date is between February 14th @ 8:00am and March 8th @ 6:00am and HbA1c ≥ 9% or FPG > 221 then Enrichment=”Enriched”.
- If subject start date is between March 8th @ 6:00am and May 8th @ 8:00am and HbA1c ≥ 11% or FPG > 275 then Enrichment=”Enriched”.
- If subject start date is between May 8th @ 6:00am and study close date then Enrichment=”Not Enriched”.

A list of subjects will be provided for those enrolled during Enrichment dates but not meeting enrichment criteria.

3. IDX_DR RESULT

- As per latest communications the final categorizations for IDx device would be provided in the final locked dataset.

4. FPRC DERIVATION ALGORITHM

The Grading Charter is as follows:

From the grading charter: DR Severity Eye	ETDRS Recode Value
10	1
12	1
14A	2
14B	2
14C	2
14Z	2
15	2
20	2
35A	3
35B	3
35C	3
35D	3
35E	3
35F	3
43A	4
43B	4
47A	5

47B	5
47C	5
47D	5
53A	6
53B	6
53C	6
53D	6
53E	6
60	7
61A	7
61B	7
65A	8
65B	8
65C	8
71A	9
71B	9
71C	9
71D	9
75	10
81	11
85A	12
85B	12
90	90

Step 1) Based on Performed Date, graders are sorted. If there are 4 graders present, the last grader data is removed.

Step 2) If there are results populated by 2 graders only:

a) If results by 2 graders are same on basis of recode values then the final grade is the highest grade as ordered in the grading charter or first grade within the recode value.
b) Exception: If the grades by 2 graders are a 10 or 12 then by default the final grade would be the one by first grader.
c) If values by 2 graders are different then 3rd grader required. A list of any such cases with a missing third grader will be provided.

Step 3) If there are results populated by 3 graders:

a) If the results by 2 graders match on the recode values then final grade is the highest grade as ordered in the grading charter or first grade within the recode value.
b) If the results by 3 graders are not matching on the recode values then the final grade is a 90. A list of the cases with a final grade of 90. The list will indicate the individual reader scores per eye to determine if the eye-level results are no/mild or mtm.
c) Note: Grade 90 will be included in the primary analysis when all reader assessments match, e.g. all no/mild or all mtm considering: 10, 12 (neither none or mild), 14A, 14B, 14C, 14Z, 15, 20 = no/mild; 35A, 35B, 35C, 35D, 35E, 35F, 43A, 43B, 47A, 47B, 47C, 47D,53A, 53B, 53C, 53D, 53E, 60, 61A, 61B, 65A, 65B, 65C, 71A, 71B, 71C, 71D, 75, 81, 85A, 85B =mtmDR
A list of all cases classified as 12 will be provided. A list of all cases classified as 60 will be provided.

Step 4) The results gathered above are bucketed into the following:

d) 10=no

e) 12=included in no/mild for primary analysis purposes
f) 14A, 14B, 14C, 14Z, 15, 20 = mild
g) 35A, 35B, 35C, 35D, 35E, 35F, 43A, 43B, 47A, 47B, 47C, 47D=moderate DR
h) 53A, 53B, 53C, 53D, 53E, 60, 61A, 61B, 65A, 65B, 65C, 71A, 71B, 71C, 71D, 75, 81, 85A, 85B =vtDR

Step 5) Considering **DME Cases**:

The 4 categories for DME cases are mapped as shown below for each eye in order of severity.

i. If "Absent" = No ME
ii. If "Questionable" = Questionable
iii. If "Cannot Grade" = cannot grade
iv. If "Definite Criteria 1 or 2" OR "Retinal Thickening or Adjacent HE < 600 microns from Center" OR "Zone of Retinal Thickening > 1 DA, part < 1 DD from Center" = Yes ME

a) If the results are populated only by 2 graders and if the results agree then the final DME is the level that they agree upon.
b) If the results are populated only by 2 graders and if the results disagree then the final DME is left missing since this would be a data issue.
c) If the results are populated by 3 graders and if the results by 2 graders agree then the final DME is the level that they agree upon.
d) If the results are populated by 3 graders and if the results by 3 graders are different then the final DME is "Cannot Grade".
A list of all cases with DME in either or both eyes will be provided.

Step 6) Combine eyes for final DME grade

After grading each eye in the order of severity, the final DME score is the most severe score between both eyes; with the understanding that 'Questionable' is worse than 'Absent' and either 'missing' or 'cannot grade' are the worst DME grades.

Step 7) Combining eyes for Diabetic Retinopathy and DME Results:

a) If there is mtmDR in either or both eye from Step 4 OR
If either or both eyes have DME from Step 6 then the final exam grade is mtmDR.
b) If the result from Step 4 is missing or No/Mild and the DME result from Step 6 is "Cannot grade" or "Questionable" then the case is excluded for Primary Analysis but included in Secondary Analysis as both ME+ and ME-.
c) If the result from Step 4 is No/Mild and the DME result from Step 6 is not equal to "Cannot grade" or "Questionable" then the final grade would be mapped to No/Mild.

Step 8) Excluding IDx insufficient image quality exams

a) If the exam had IDx insufficient image quality, subject is excluded.
b) If the exam had sufficient image quality, subject is included for primary analysis

5. <u>IDX IMPUTATION ALGORITHM</u>

a) Best case scenario

As per SAP where all missing IDx-DR results, including insufficient exam quality and technical failures, completely agree with the FPRC reference results.

- If the IDx-DR result is missing and FPRC result ="no/mild" then the imputation will be "No mtmDR"
- If the IDx-DR result is missing and FPRC result ="mtmDR" then the imputation will be "mtmDR"

b) Worst case scenario

As per SAP where all missing IDx-DR results, including insufficient exam quality and technical failures, completely disagree with the FPRC reference results.

- If the IDx-DR result is missing and FPRC result =”no/mild” then the imputation will be “mtmDR”
- If the IDx-DR result is missing and FPRC result =”mtmDR” then the imputation will be “No mtmDR”.

c) Derivations

TPmtm (A)	IDx-DR “mtmDR Detected” AND FPRC grade of 35A, 35B, 35C, 35D, 35E, 35F, 43A, 43B, 47A, 47B, 47C, 47D, 53A, 53B, 53C, 53D, 53E, 60, 61A, 61B, 65A, 65B, 65C, 71A, 71B, 71C, 71D, 75, 81, 85A, 85B and/or Macular Edema Present
TNn/m (B)	IDx-DR “Negative for mtmDR” AND FPRC grade of 10, 12, 14A, 14B, 14C, 14Z, 15, 20
FNmtm (C)	IDx-DR “Negative for mtmDR” AND FPRC grade of 35A, 35B, 35C, 35D, 35E, 35F, 43A, 43B, 47A, 47B, 47C, 47D, 53A, 53B, 53C, 53D, 53E, 60, 61A, 61B, 65A, 65B, 65C, 71A, 71B, 71C, 71D, 75, 81, 85A, 85B and/or Macular Edema Present
FPn/m (D)	IDx-DR “mtmDR Detected” AND FPRC grade of 10, 12, 14A, 14B, 14C, 14Z, 15, 20

Note: per the SAP, only results with a diagnostic output from IDx-DR and a Reading Center result will be included in primary analysis, assuming IDx-DR rejects less than 10% of exams for insufficient quality photos.

For secondary analyses, the following definitions will apply:

TPmtm (A)	IDx-DR “mtmDR Detected” AND FPRC grade of 12*, 35A, 35B, 35C, 35D, 35E, 35F, 43A, 43B, 47A, 47B, 47C, 47D, 53A, 53B, 53C, 53D, 53E, 60, 61A, 61B, 65A, 65B, 65C, 71A, 71B, 71C, 71D, 75, 81, 85A, 85B or Macular Edema Present
TNn/m (B)	IDx-DR “Negative for mtmDR” AND FPRC grade of 10, 12*, 14A, 14B, 14C, 14Z, 15, 20
FNmtm (C)	IDx-DR “Negative for mtmDR” AND FPRC grade of 12*, 35A, 35B, 35C, 35D, 35E, 35F, 43A, 43B, 47A, 47B, 47C, 47D, 53A, 53B, 53C, 53D, 53E, 60, 61A, 61B, 65A, 65B, 65C, 71A, 71B, 71C, 71D, 75, 81, 85A, 85B and/or Macular Edema Present
FPn/m (D)	IDx-DR “mtmDR Detected” AND FPRC grade of 10, 12*, 14A, 14B, 14C, 14Z, 15, 20
IDx-DR imputed TPmtm (E) or FNmtm (F)	IDx-DR Insufficient AND FPRC Result of 35A, 35B, 35C, 35D, 35E, 35F, 43A, 43B, 47A, 47B, 47C, 47D, 53A, 53B, 53C, 53D, 53E, 60, 61A, 61B, 65A, 65B, 65C, 71A, 71B, 71C, 71D, 75, 81, 85A, 85B
IDx-DR imputed TNn/m (G) or FPn/m (H)	IDx-DR Insufficient AND FPRC Result of 10, 12, 14A, 14B, 14C, 14Z, 15, 20
No Gold Standard	No FPRC Result available

Imaging (W)	
No FPRC Truth (X)	FPRC final result of 90 after completing all Steps in Section 5 of the BDRM Prep Note to File (No. 5)
No Gold Standard Imaging (Y)	No FPRC Result available
No FPRC Truth (Z)	FPRC final result of 90 after completing all Steps in Section 5 of the BDRM Preparation Note to File (No. 5)

As noted in the SAP, the special case of ETDRS 12s will be considered in an important secondary endpoint analysis that will recognize the challenge of ruling out the presence of DR in eyes where incidental abnormalities are present.

As described by the FPRC in their logged Note to File, in the FPRC Grading and Imaging Charter for IDx-DR1, FPRC pre-specified that eyes receiving a score of ETDRS 10 (i.e., no disease) and ETDRS 12 (i.e., an incidental finding, potentially precluding a severity-level grading of diabetic retinopathy) would be at the same "recode" level. This is because while one grader noted an incidental finding, ultimately, both graders agreed no diabetic retinopathy could be characterized using the broader ETDRS scale.

Based on this understanding, a key secondary analysis will include those 12's where at least some graders could make a DR severity level finding and removes those 12's where it is not clear whether the graders could rule out the presence of DR. Repeating our topline analysis after removing the exams where a consensus ETDRS 12 is the most severe grade will provide an alternate evaluation of IDx-DR's performance that accommodates the unique case of ETDRS 12s, while still preserving as many study cases as possible.

Table: ETDRS 12 Disposition

Analysis Description	Specific Action
ETDRS consensus 12's removed	Repeat topline analysis after removing the exams where a consensus ETDRS 12 is the most severe grade.<br><br>12's that remain in this secondary analysis: 10-12, 12-10, 12-[XX]-[XX] (where XX = XX ≠ 12), 12-[XX]-[YY] (where XX ≠ YY AND XX ≥ 35 AND YY ≥ 35)<br><br>12's that are removed from this secondary analysis: 12-12, 12-[>12]-12, and 12-[XX]-[YY] (where XX ≠ YY AND EITHER XX < 35 OR YY < 35)

Primary Endpoint Calculations:

Sensitivity will be evaluated with and without imputations using the following calculations:
mtm Sensitivity: A/(A+C)

Specificity will be evaluated using the logistic regression model described in section 8.

Additional analysis for cases without reading center truth:
Best cases:
TP= (A+W+X)/ (A+C+W+X)

TN=D/ (D+B+W+X)
Worst cases:
FN=1-TP
FP=1-TN

Secondary Endpoint Calculations:

Insufficient image quality treated as mtmDR+
mtm Sensitivity: (A+E)/(A+C+E)
mtm specificity: D/(D+B+H)

Insufficient image quality treated as no/mild
mtm Sensitivity: (A)/(A+C+F)
mtm specificity: (D+G)/(D+B+G)

Insufficient image quality treated as 100% agreement/standard (best case)
mtm Sensitivity: (A+E)/(A+C+E)
mtm specificity: (D+G)/(D+B+G)

Insufficient image quality treated as 100% disagreement/standard (worst case)
mtm Sensitivity: (A)/(A+C+F)
mtm specificity: (D)/(D+B+H)

6. FPRC EXCLUSIONS

The FPRC categorical results are populated as per approach mentioned in No 5 above. Based on this,

- If there is Insufficient Exam Quality the subject is excluded in both Primary and Secondary Analysis.
- If the final FPRC score is a 12, the subject is included in Primary Analysis, and consensus 12s will be handled per Secondary Analyses rules.
- If the Final FPRC score is a 60 the subject is included in primary analysis and excluded for Secondary Analysis.
- If the Final FPRC score is DME Questionable the subject is excluded from primary analysis and imputed as positive and negative for Secondary Analyses.

7. PRIMARY EFFECTIVENESS ENDPOINTS

a) Sensitivity and Specificity

Sensitivity and Specificity provides us details on how good the IDx Device is when compared to the FPRC standard results. One-sided 97.5% confidence intervals and p-value for the lower bounds will also be calculated for these binary measures.

- Subjects with mtmDR counts towards the sensitivity numerator.
  Ratio of subjects diagnosed positive by the IDx Device to the subjects diagnosed positive by the FPRC.

- Subjects with No/Mild mtmDR counts towards specificity numerator.
  Ratio of subjects diagnosed negative by the IDx Device to the subjects diagnosed negative by the FPRC.

- In calculating the mtmDR specificity, the estimation analyses will be carried out on the combined cohorts. The impact of the following baseline covariates will be considered to evaluate cohort effect using a logistic regression model using the Firth method to compute one-sided 97.5% lower confidence intervals for specificity vs >7%).

8. SECONDARY EFFECTIVENESS ENDPOINTS

a) Positive Predictive Value & Negative Predictive Value:

PPV and NPV provides us details on how the FPRC results can be graded on basis of our IDx device.
One-sided lower 97.5% confidence intervals and corresponding p-values will also be calculated for these binary measures.

Analyses will be run for the ITS population with and without imputation.

- Ratio of subjects diagnosed positive by the FPRC to the subjects diagnosed positive by the IDx Device gives PPV.
- Ratio of subjects diagnosed negative by the FPRC to the subjects diagnosed negative by the IDx Device gives NPV.

b) Positive Likelihood Ratio & Negative Likelihood Ratio:

PLR and NLR are derived on the basis of sensitivity and specificity.
One-sided 97.5% confidence intervals for the lower bounds will also be calculated for these binary measures.

- PLR=Sensitivity/1-Specificity
- PLR=1-Sensitivity/Specificity

c) Impact of Baseline Characteristics on Sensitivity and Specificity

Results will be reported with Coefficient, Standard Error, p-Value, Odds Ratio, corresponding one-sided 97.5% confidence intervals (CI) and Area under the ROC curve which gives final Decision.

d) In calculating the mtmDR sensitivity, the estimation analyses will be carried out on the combined cohorts. The impact of the following baseline covariates will be considered to evaluate cohort effect using a logistic regression model using the Firth method to predict sensitivity: age (<65 yrs and ≥65 yrs), gender, race (black vs Caucasian; other vs Caucasian), ethnicity (Hispanic/Latino vs Non-Hispanic/Latino), HbA1c (<9% vs >9%).

9. ENDPOINT ANALYSES

Primary and secondary analysis will be performed on the following:

a) All qualifying cases included, 0.11 algorithm
b) Excluding EDTRS 12 per the ETDRS 12 Disposition Table (specificity) and 60 (sensitivity), 0.11 algorithm
c) Best case, all qualifying, 0.11 algorithm
d) Worst case, all qualifying, 0.11 algorithm

Additionally as supplemental analyses, these analyses will also be performed using for a 0.07 imaging algorithm threshold for the mtmDR algorithm in order to illustrate a different configuration of the algorithm. This threshold is one that falls within the range of validated configurable set points allowed in the EU version of IDx-DR, which can be deployed to be more sensitive to disease detection based on clinical preference.

Appendix 2: Addendum to SAP Supplement, November 29, 2017

This addendum is needed following test data runs to check logistic regression model convergence for the pre-specified covariates identified in the SAP Supplement dated November 10, 2017.

It was found that the models took too long to converge so an alternative plan was developed prior to database lock. The problem arose from covariates that took on the same binary value for >95% of all study subjects.

Solution

We agreed to not run the Firth method on the full logistic regression model containing the full list of baseline covariates.

To simplify clarification, FPRC O corresponds to the subjects classified as none or mild by FPRC while FPRC 1corresponds to the subjects classified as moderate or vt DR by FPRC. There is also an enriched vs not enriched covariate. The dependent variable is always the binary outcome.

To address the problem and to anticipate possible requests from FDA, we will Instead run the following with the first being the primary analysis for specificity:

A logistic regression model of IOx=Enriched (n,y) for just FPRC=0 subjects (No/mild DR by FPRC)

A logistic rgression model of IOx= Enriched (n,y) for just FPRC=1 subjects (mtm DR by FPRC)

A logistic regression model of IOx=Covariates for just FPRC=0 subjects

A logistic regression model of IOx=Covariates for just FPRC=1 subjects

A logistic regression model of IDx=FPRC containing all subjects.

The third and fourth models correspond to the SAP supplement.

We will use Firth to make the corrected point estimates and corresponding one-sided 97.5% lower bounds.
