# Supplementary material for: Pivotal trial of an autonomous AI-based diagnostic system for detection of diabetic retinopathy in primary care offices
Source: NPJ Digit Med. 2018 Aug 28;1:39. doi: 10.1038/s41746-018-0040-6 (PMC6550188; doi:10.1038/s41746-018-0040-6)
Supplement: Supplementary file 2 — IDx-DR pivotal study SAP Supplemental Tables 1 and 2 [file 41746_2018_40_MOESM2_ESM.pdf]

IDX-DR1 PROTOCOL SYNOPSIS

IDX, LLC
Clinical Study Protocol

TITLE: A MULTICENTER STUDY TO EVALUATE PERFORMANCE OF AN AUTOMATED DEVICE FOR THE DETECTION OF DIABETIC RETINOPATHY

Protocol Number:	IDx-DR1
Version:	2 (Synopsis)
Version Date:	August 17, 2017
Investigational Product:	IDx-DR
IDE Number:	IDE exempt; subject to abbreviated requirements under 21 CFR 812.2(b)
Sponsor:	IDx, LLC<br>485 Hwy 1 W<br>Iowa City IA 52246
Funding Organization:	IDx, LLC
Regulatory Consultant	Janice Hogan – Hogan Lovells
Study Statistician:	Phil Lavin, PhD – Lavin Consulting LLC
Contract Research Organization:	Emmes Corporation
Photographic Reading Center:	The University of Wisconsin Fundus Photograph Reading Center
Study Chair & Medical Monitor:	Michael Abramoff, MD, PhD

PROTOCOL SYNOPSIS

**TITLE**	**A MULTICENTER STUDY TO EVALUATE PERFORMANCE OF AN AUTOMATED DEVICE FOR THE DETECTION OF DIABETIC RETINOPATHY**
**SPONSOR**	IDx LLC
**NUMBER OF SITES**	Up to 15
**RATIONALE**	Diabetes affects 29.1 million people or 9.3% of the population of the United States. Results of a study by the Eye Diseases Prevalence Research Group reveal that 40% of diabetes patients have some degree of diabetic retinopathy (DR) and that as many as 8% have severe, vision-threatening forms of DR. Early laser photocoagulation in high-risk proliferative diabetic retinopathy (PDR) has been shown to decrease the relative risk of vision loss by as much as 52%. Injections of anti-VEGF agents in multiple clinical trials have been shown to preserve and improve vision in people with PDR and/or diabetic macular edema. Despite effective treatment however, tens of thousands of people with diabetes are going blind each year largely because they don't undergo annual screening for retinopathy. Currently, only about 60% of people with diabetes have a yearly eye exam and there may not be enough eye specialists to see the balance.<br><br>To address the issue of patient compliance with diabetic retinopathy screening, IDx-DR was developed as an automated screening device designed to analyze fundus images for the presence of lesions and other disease features associated with diabetic retinopathy. This study has been designed to validate the safety and efficacy of the device at the frontlines of healthcare.
**STUDY DESIGN**	Multicenter Observational Study
**DEVICE**	IDx-DR is a software device intended for use by health care providers to screen for more than mild diabetic retinopathy (mtmDR) in adult (22 years of age or older) people with diabetes who have not been previously diagnosed with diabetic retinopathy. The device is indicated for use with fundus photographs obtained by the Topcon NW400. A result of mtmDR indicates a high risk of moderate non-proliferative retinopathy, severe non-proliferative retinopathy (NPDR), proliferative retinopathy (PDR), and/or macular edema.
**PRIMARY OBJECTIVE**	To demonstrate the sensitivity and specificity of IDx-DR's automated image analysis for mtmDR when used by health care providers to screen for mtmDR in adults (22 years of age or older) diagnosed with diabetes who have not been previously diagnosed with diabetic retinopathy.
**NUMBER OF SUBJECTS**	845 subjects after exclusions (inclusive of 189 mtmDR subjects and 656 no/mild subjects)

**TITLE**	**A MULTICENTER STUDY TO EVALUATE PERFORMANCE OF AN AUTOMATED DEVICE FOR THE DETECTION OF DIABETIC RETINOPATHY**
**ENDPOINTS**	Sensitivity and Specificity of IDx-DR, analysis per SAP
**SUBJECT ELIGIBILITY CRITERIA**	Participant Inclusion Criteria:<br>1. Documented diagnosis of diabetes mellitus, e.g.:<br>a. Having met the criteria established by either the World Health Organization (WHO) or the American Diabetes Association (ADA)<br>b. Hemoglobin A1c (HbA1c) ≥ 6.5%<br>c. Fasting Plasma Glucose (FPG) ≥ 126 mg/dL (7.0 mmol/L)<br>d. Oral Glucose Tolerance Test (OGTT) with two-hour plasma glucose (2-hr PG) ≥ 200 mg/dL (11.1 mmol/L), using the equivalent of an oral 75 g anhydrous glucose dose dissolved in water<br>e. Symptoms of hyperglycemia or hyperglycemic crisis with a random plasma glucose (RPG) ≥ 200 mg/dL (11.1 mmol/L)<br>2. Age 22 or older<br>3. Understand the study and volunteer to sign the informed consent<br><br>Participant Exclusion Criteria:<br>1. Persistent vision loss, blurred vision, or floaters.<br>2. Diagnosed with macular edema, severe non-proliferative retinopathy, proliferative retinopathy, radiation retinopathy, or retinal vein occlusion.<br>3. History of laser treatment of the retina or injections into either eye, or any history of retinal surgery.<br>4. Currently participating in another investigational eye study and actively receiving investigational product for DR or DME.<br>5. Participant has a condition that, in the opinion of the investigator, would preclude participation in the study (e.g., unstable medical status including blood pressure or glycemic control, microphthalmia or previous enucleation).<br>6. Participant is contraindicated for imaging by fundus imaging systems used in the study:<br>o Participant is hypersensitive to light<br>o Participant recently underwent photodynamic therapy (PDT)<br>o Participant is taking medication that causes photosensitivity

I. BACKGROUND

Diabetic retinopathy (DR) is the most common cause of blindness in the working population of the United States [1]. Each year, DR leads to more than 24,000 cases of preventable blindness in the United States. An estimated 4.1 million and 899,000 Americans are affected by retinopathy and vision-threatening retinopathy, respectively [2].

Early detection of DR can prevent vision loss and blindness [3-7]; however, a large portion of individuals with diabetes do not currently undergo any DR screening. With the prevalence of diabetes expected to increase 50% over the next 10 years, screening rates, detection, and treatment must be addressed in order to prevent a significant increase in unnecessary blindness across the United States [8-10].

A recent publication [11] reviewed the key research related to eye screening rates for people with diabetes, and concluded that screening rates remain poor. A 1990 program launched by the American Academy of Ophthalmology (AAO), known as Diabetes 2000, was designed to increase nationwide DR screening rates. However, after 20 years of implementation, screening rates continue to stagnate, with only 50-60% of diabetic patients properly evaluated [11]. In addition, specific disadvantaged groups have increased prevalence of diabetic retinopathy, where low rates of DR screening are at least partially associated with lack of access to a specialist exam [12]. A DR screening solution at the point of primary care is expected to help address the unmet need, lack of access, and racial and ethnic disparity issues that have persisted for decades.

Some improved compliance with AAO defined screening recommendations has already been enabled by telemedicine, a practice associated with reduced incidence of blindness [13]. In a recent prospective randomized study of telemedicine, availability of telemedicine resulted in a 50% increase in screening compliance [14]. However, implementation of telemedicine requires readers, who introduce variability across inter-observer and intra-observer measures. Telemedicine also presents issues with training and reader turnover, economic feasibility challenges, IT infrastructure barriers, as well as the potential for substantial delay between imaging and the availability of results (often a time period of days or weeks) [15].

An automated screening device would allow for immediate screening results at the point of care, standardization of results, and elimination of most overhead costs associated with telemedicine. Leaders in the ophthalmic community report that an automated diabetic retinopathy screening device in the hands of front line providers would increase screening rates and detect more treatable vision threatening retinopathy [14].

II. STUDY RATIONALE

Diabetes affects 29.1 million people or 9.3% of the population of the United States and there are 1.4 million new cases of diagnosed diabetes each year. In 2012, 86 million Americans age 20 and older had prediabetes; this was up from 79 million from 2010 [16]. Results of a study by the Eye Diseases Prevalence Research Group reveal that 40% of diabetes patients have some degree of DR and that as many as 8% have severe, vision-threatening forms of DR. Early laser photocoagulation in high-risk proliferative diabetic retinopathy (PDR) has been shown to decrease the relative risk of vision loss by as much as 52%. Injections of anti-VEGF agents preserve and improve vision in people with PDR and/or diabetic macular edema. Despite effective treatment, however, tens of thousands of people with diabetes are going blind each year largely because they do not undergo annual screening for retinopathy. Currently, only about 50%-60% of people with diabetes have a yearly eye exam and there may not be enough eye specialists to see the balance [10, 17-19].

To address the issue of patient compliance with diabetic retinopathy screening, IDx-DR offers an automated image interpretation device that enables providers at the frontlines of healthcare to increase access to and reduce the cost of screening. This study is designed to assess the sensitivity and specificity of the IDx-DR device in screening for more than mild diabetic retinopathy (mtmDR).

III. STUDY OVERVIEW

The study is a multicenter observational study with primary endpoints to determine the sensitivity and specificity of IDx-DR for detection of mtmDR in the primary care setting. IDx-DR is an automated software device that is designed to analyze ocular fundus digital color photographs taken in primary care settings in order to quickly screen for mtmDR.

Participants who meet the eligibility criteria will be recruited from sites staffed by primary care providers. After assessing eligibility and securing written informed consent, fundus photographs will be captured using a commercially available, Food and Drug Administration (FDA) cleared, non-mydriatic ocular fundus camera – the Topcon NW400 (NW400) fundus camera – by a camera operator who does not have previous professional experience in ophthalmic photography. Images will be taken according to a specific IDx-DR imaging protocol provided to the NW400 camera operator, and then analyzed by the IDx-DR device.

The photography protocol, as described below, consists of two images of the ocular fundus (one optic disc centered, one fovea centered), obtained from both eyes of enrolled participants using the NW400. If the NW400 camera operator is unable to obtain adequate images without dilation, the participant will be asked to remove contact lenses if they are wearing contacts and undergo re-imaging under dilation using tropicamide 1% eye drops, administered under the supervision of a physician. After generating an IDx-DR screening result, a separate professional ophthalmic photographer certified by the Wisconsin Fundus Photography Reading Center (FPRC) will use a different, FDA-cleared camera system called the Topcon 3D OCT-1 Maestro (Maestro) to perform dilated W-4 four-field fundus photography, macular ocular coherence tomography (OCT) imaging, and imaging for the grading of media opacity. W-4 and OCT images using the Maestro will be sent to FPRC for primary and secondary reference standard evaluation.

The FPRC will determine the severity of retinopathy (including the presence of macular edema) according to the Early Treatment of Diabetic Retinopathy System (ETDRS) grading system based on the W-4 fundus photographs. OCT will also be used to assess the presence of diabetic macular edema (DME) according to FPRC grading protocols. Incidental findings will also be documented by the FPRC, including review of grading for media opacity.

In order to assure an adequate range of DR conditions in the study population for sensitivity and specificity analysis, the study population will be enriched with participants at higher risk for vtDR. The enrichment cohort will be targeted using HbA1c and/or FPG levels, if needed.

The performance of the IDx-DR device will be compared to the FPRC readings in terms of sensitivity and specificity, in addition to conducting stratification based on media opacities and participant demographics, including ethnicity and race, cataract, incidental findings, and referral attrition. Endpoints will be calculated according to the Statistical Analysis Plan (SAP).

IV. CRITERIA FOR EVALUATION

The primary efficacy endpoints in the study are to demonstrate that IDx's intended image capture workflow – in combination with the IDx-DR device – can successfully be used to provide a diagnostic result.

V. DEVICE SAFETY EVALUATION

The proposed trial is expected to introduce only minimal risk to both NW400 camera operators and participants because the camera systems being used have previously been cleared by the FDA for ocular fundus photography and tropicamide 1% eye drops for pupil dilation are an FDA-approved drug product that has been demonstrated to have minimal side effects[20]. Ongoing safety monitoring will be performed by the Contract Research Organization (CRO), who will review reported adverse effects (AEs) or unanticipated adverse device effects (UADEs) received from clinical trial sites on a regular basis and report them promptly in communications to the Sponsor.

VI. CLINICAL ASSESSMENTS

A. Demographics

Demographic information collected from participants as part of this study will include birth date, sex, ethnicity and race. Per the consent form and protocol, no additional identifiable participant data will be recorded for study purposes.

B. Medical Assessment

Participants in the study will be assessed at the primary care site for the purpose of determining eligibility. This assessment includes a review of medical records and questions asked by primary care personnel or clinical study staff. Participants will be confirmed for a diagnosis of diabetes by testing or a review of medical history, and will be asked whether they have persistent vision loss, blurred vision, or floaters. They will also be asked if they are contraindicated for fundus photography on the Topcon NW400 on the basis of hypersensitivity to light, recent photodynamic therapy (PDT), or taking of medication that causes photosensitivity. These terms will be explained as part of the eligibility determination process and in the informed consent document. Participants will be asked whether they are diagnosed with macular edema, severe non-proliferative retinopathy, proliferative retinopathy, radiation retinopathy, or retinal vein occlusion. They will also be asked if they have any history of laser treatment of the retina or retinal surgery (including intraocular injections). The site may also review the participant’s records for the same information. The initiation, continuation or discontinuation of medical therapy will remain at the discretion of the Investigator, as this is not expected to be dependent on any aspect of participation in this research study.

C. Reading Center Assessment and CRO Follow-Up

The Reading Center will be providing an EDTRS result for each participant in the study within 2-10 weeks of their completion of the study protocol.

To ensure patients are appropriately referred to an eye care professional based on Reading Center grades, all adjudicated ETDRS Reading Center results will be converted to an ICDR level by the CRO based on the American Academy of Ophthalmology’s (AAO) International Clinical Diabetic Retinopathy (ICDR) Disease Severity Scale (http://www.icoph.org/downloads/Diabetic-Retinopathy-Detail.pdf). ICDR results provided to sites will also indicate whether the Reading Center detected macular edema (ME).

To support referral determinations based on ICDR results provided to them by the CRO, participating clinicians should review the Management Recommendations for

Patients with Diabetes as contained in the AAO's Preferred Practice Pattern (AAO PPP) Recommendations for Diabetic Retinopathy (See Table 6 at http://www.aao.org/preferred-practice-pattern/diabetic-retinopathy-ppp-updated-2016).

TABLE 6 MANAGEMENT RECOMMENDATIONS FOR PATIENTS WITH DIABETES

Severity of Retinopathy	Presence of Macular Edema	Follow-up (Months)	Panretinal Photocoagulation (Scatter) Laser	Focal and/or Grid Laser*	Intravitreal Anti-VEGF Therapy
Normal or minimal NPDR	No	12	No	No	No
Mild NPDR	No	12	No	No	No
	ME	4–6	No	No	No
	CSME†	1*	No	Sometimes	Sometimes
Moderate NPDR	No	12‡	No	No	No
	ME	3–6	No	No	No
	CSME†	1*	No	Sometimes	Sometimes
Severe NPDR	No	4	Sometimes	No	No
	ME	2–4	Sometimes	No	No
	CSME†	1*	Sometimes	Sometimes	Sometimes
Non-high-risk PDR	No	4	Sometimes	No	No
	ME	2-4	Sometimes	No	No
	CSME†	1*	Sometimes	Sometimes	Sometimes
High-risk PDR	No	4	Recommended	No	Alternative[129,130]
	ME	4	Recommended	Sometimes	Usually
	CSME†	1*	Recommended	Sometimes	Usually

Anti-VEGF = anti-vascular endothelial growth factor; CSME = clinically significant macular edema; ME = non-clinically significant macular edema; NPDR = nonproliferative diabetic retinopathy; PDR = proliferative diabetic retinopathy

* Adjunctive treatments that may be considered include intravitreal corticosteroids or anti-VEGF agents (off-label use, except aflibercept and ranibizumab). Data from the Diabetic Retinopathy Clinical Research Network in 2011 demonstrated that, at two years of follow-up, intravitreal ranibizumab with prompt or deferred laser resulted in greater visual acuity gain and intravitreal triamcinolone acetonide plus laser also resulted in greater visual gain in pseudophakic eyes compared with laser alone.[131] Individuals receiving the intravitreal injections of anti-VEGF agents may be re-examined as early as one month following injection.

† Exceptions include hypertension or fluid retention associated with heart failure, renal failure, pregnancy, or any other causes that may aggravate macular edema. Deferral of photocoagulation for a brief period of medical treatment may be considered in these cases.[132] Also, deferral of CSME treatment is an option when the center of the macula is not involved, visual acuity is excellent, close follow-up is possible, and the patient understands the risks.

‡ Or at shorter intervals if signs approaching those of severe NPDR appear.

In correspondence about Reading Center results, clinicians will also be provided with the following notes regarding the PPP:

- The AAO PPP recommends a 12-month follow-up for patients with normal, minimal non proliferative diabetic retinopathy (NPDR) or mild NPDR when there is no presence of macular edema (ME).
- The AAO PPP recommends that it may be appropriate for patients with moderate NPDR to receive follow-up examination by an eye care professional in an interval less than 12 months.

- Based on the AAO PPP, those patients with severe NPDR, Non-high risk PDR, or High Risk PDR – as well as those with macular edema – may be indicated for treatment based on evaluation by an eye care professional. These subjects should be referred.

On the basis of Reading Center ICDR results and AAO PPP Recommendations, physicians will be able to provide appropriate eye care referrals for participants.

VII. CLINICAL SITE COMMUNICATION, MANAGEMENT AND MONITORING

A. Site Monitoring

The CRO will follow standard operating procedures for monitoring this study in accordance with Good Clinical Practice (GCP) recommendations and FDA regulatory requirements. Any site not meeting the minimum requirements to initiate the trial, or that has administrative, procedural or data quality deficiencies that require correction in order to comply with regulatory requirements, the protocol, or to meet the requirements of the sponsor and the CRO, will be notified in writing of the deficiencies and permitted a reasonable opportunity to rectify deficient conditions. After study initiation, the inability of the site to rectify seriously deficient conditions in a timely manner or to maintain compliance with regulatory requirements may be cause for termination of study activities, closure of the investigational site, and notification of that decision to the relevant Institutional Review Board (IRB) or Independent Ethics Committee (IEC) and other regulatory authorities as appropriate.

Participating sites will have an initial monitoring visit, routine interim monitoring visits (at a schedule determined by sponsor or CRO) during the study and a study close-out visit conducted by experienced monitoring personnel. Additional monitoring visits may be performed for cause or if the volume of information to be reviewed cannot be easily completed in a single visit. Study visits will normally be scheduled well in advance so that necessary site staff and appropriate records will be available during the monitoring visit.

Each monitoring visit will utilize a standardized checklist of elements to be reviewed at the site, tailored to the specific requirements of this study. Site monitoring visits will routinely review the participating site staff roster; study administrative and financial documents; required regulatory documentation; status of IRB/IEC approvals; changes or actions taken since any previous visit; participant recruitment status, screening, enrollment, and follow-up visit records; documentation of informed consent for each participant; review of adverse events; investigational product storage conditions; outstanding data clarifications and a review of data elements against source documentation. Site visits will follow standard CRO procedures and a report will be prepared for study records.

B. Essential Document Management

The CRO will obtain and maintain essential regulatory documentation for the clinical trial investigator sites only to the extent described in the International Conference on Harmonization (ICH) Good Clinical Practice (GCP) guidelines and 21 CFR 812.140(a), and those Sponsor records listed under 812.140(b) only if they are provided to the CRO by the Sponsor. The regulatory documentation does not contain

clinical records, CRFs or other source documentation regarding individual participants.

The site Principal Investigator is required to maintain this documentation in a suitable regulatory binder or similar filing system and make it available for review by authorized study monitors, auditors and regulatory authorities. The Sponsor or their designee will maintain a master trial file. Both current and outdated study documents must be maintained. Older versions of documents may be stored elsewhere in a secure location, provided a reference to the actual storage location remains in the binder. If the binder contents become voluminous, multiple volumes may be maintained. The site Principal Investigator or Study Coordinator/Research Contact maintains the Regulatory Binder. Contents of the regulatory binder(s) or master trial file must include the following sections (the order of which may vary), which may be supplemented with additional dividers/folders if necessary. Any empty or unused sections should contain a document acknowledging this status.

Regulatory Binder/Master Trial File

a. Investigator Agreement
b. Curriculum vitae and other qualification documents (licenses, training) if applicable
c. Financial disclosure and/or Conflict of Interest certifications
d. Regulatory (FDA) authority authorization (where applicable)
e. IRB/IEC approvals
f. IRB roster or Letter of Assurance
g. Approved Clinical Protocol and any amendments
h. Approved Informed Consent
i. Participant recruitment materials and any other written information given to Participants (where applicable)
j. Investigator’s Brochure (or relevant product labeling)
k. Safety Reports (from other sites or sponsor)
l. Serious Adverse Event summaries (from this site)
m. Laboratory certifications and normal reference ranges for any tests required by the protocol, unless the test method is CLIA-waived
n. Authorized site staff signature log documenting authorized study responsibilities
o. Site Visit Monitoring Log
p. All monitoring reports including the final trial close-out monitoring report
q. Correspondence

The CRO will track submission of the study protocol and informed consent documents to the IRB/IEC responsible for each site using the Emmes Regulatory Tracking System (RTS), and notify the sponsor when documented approval or favorable opinion from the IRB/IEC has been obtained for each clinical trial site. The CRO will also track signed, dated, and completed Case Report Forms (CRF’s) if paper forms are used. Sites are responsible for maintaining signed informed consent forms and the participant enrollment log.

C. Data Management and Transfer

The CRO will host and manage a web-based integrated clinical data system called Advantage eClinical according to their SOPs and in compliance with FDA’s guidance on Computerized Systems Used in Clinical Investigations (2007) and 21 CFR Part 11. The CRO will also develop and implement standard web-based electronic Case Report Forms (eCRFs) used by the clinical trial sites and the image reading center, FPRC. Quality assurance and selected data integrity controls will be built into each eCRF, and the CRO may apply additional data integrity checks to assist in

monitoring and improving data quality. The CRO will obtain IDx-DR results directly from the computer server system that is isolated from sponsor access during and after the study until the analyses are completed according to the SAP. The CRO will securely transfer data and documentation monthly to both the FPRC and the study statistician in accordance with this protocol and Sponsor requirements. During monthly data transfers, FPRC data and IDx-DR results will never be compared; reconciliation of results will only occur at the one-time futility analysis as described below and in the final analyses.

D. Safety Monitoring

The CRO will provide safety reporting services, including review, assessment, documentation, and reporting of AEs or UADEs submitted by clinical investigators. Investigators and the Sponsor will be notified if unreported AEs or UADEs are observed in study records by the monitors during routine or for cause monitoring activities.

Events considered serious and unexpected by the site staff or the CRO will be immediately investigated by the CRO or Sponsor to determine if the event qualifies for expedited reporting to regulatory authorities. The CRO will be responsible for coordinating the expedited reporting of serious and unexpected AEs or UADEs to regulatory authorities within the required timeframes once the determination for reporting has been made. The CRO will review summary information on all reports of unexpected serious AEs or UADEs submitted by investigators, and will review individual events if the situation requires.

1. Methods and Timing for Assessing, Recording, and Analyzing Safety Parameters

Safety will be evaluated during the time the participant is in the study which will include from the time of consent through the time of exit which will typically be less than one day. Safety will be assessed by frequency and incidence of ADEs and UADEs.

Because this is considered a device study, it is consistent to use the following definitions when assessing safety issues:

- **Adverse device effect (ADE)**, which means an adverse event related to the use of an investigational medical device.
- **Unanticipated adverse device effect (UADE)**, which means any serious adverse effect on health or safety or any life-threatening problem or death caused by, or associated with its use that had not been previously identified in nature, severity, or degree of incidence.

2. Identification of Events and Timeframe for Reporting

For the purpose of this study, ADEs and UADEs will only be recorded for the duration of the participant’s involvement in the study, which will be less than one day. Many participants in this study may have pre-existing medical conditions however, those pre-existing conditions will not be considered as ADE/UADEs, unless use of the study device elicits a new ADE/UADE or worsens an existing condition in terms of frequency or intensity during or immediately after the use of the study device. All reportable events as defined above, determined to be an ADE/UADE will be recorded in the source documents and entered in the electronic case report form (e-CRF). The investigator will provide the date of onset and resolution, intensity, frequency,

action(s) taken, changes in study device application, relationship to study device, and outcome.

3. Follow-up of Adverse Device Effects

ADEs that occur on the day of the visit will be followed up to adequately evaluate the participant's safety or until the event stabilizes. When the event resolves, a resolution date should be documented on the case report form. All UADEs will be followed until resolution or until the participant is medically stable. All other events that cannot be resolved by 30 days after the last study contact will be considered resolved by convention and entered in the electronic data capture (EDC) system.

4. Guidelines for Assessing Intensity of an Adverse Device Effect

The investigator should use the following definitions when assessing intensity of an adverse event:

- MILD: Participant is aware of symptoms or has minor findings but tolerates them well, and no or minimal intervention required
- MODERATE: Participant experiences enough symptoms or findings to require intervention
- SEVERE: Participant experiences symptoms or findings that require significant medical intervention

5. Guidelines for Determining Causality

The investigator will use the following question when assessing causality of an ADE/UADE to study device, where an affirmative answer designates the event as a suspected ADE: Is there a reasonable possibility that the device or its use caused the event? ''Reasonable possibility'' means that there is evidence to suggest a causal relationship between use of the device and the ADE.

6. Discontinuation Due to ADEs/UADEs

Participants may be withdrawn from the study at any time. Participants withdrawn from the study due to an ADE, whether serious or non-serious, must be followed by the investigator until the clinical outcome from the ADE is determined. Any participant who experiences an ADE may be withdrawn at any time from the study at the discretion of the investigator. The ADE(s) should be noted on the appropriate CRFs, and the participant's progress should be followed until the ADE is resolved. The medical monitor or project manager must be notified.

7. Reporting Procedures

All recordable ADEs will be entered into the safety data system within 7 days of identification. Recordable UADEs will be entered into the data system within 24 hours of identification. If there are any technical difficulties, the UADE will be reported by telephone or fax communication. This data will be entered into the EDC as soon as the system is available.

8. Unanticipated Adverse Device Effects

Any UADE entered in the safety database by a site investigator will generate an automatic email notification to the sponsor IDx-DR Medical Monitor. The sponsor is obligated to investigate UADE reports promptly to determine reporting requirements of the event and assess if the event warrants further action by the sponsor.

9. Regulatory Reporting

Any event that may be considered a UADE related to the IDx-DR device and therefore may require reporting based on federal regulations will be forwarded to the sponsor for investigation. After assessing reporting requirements of the event, the sponsor or its representative will submit reports to the FDA and other regulatory agencies as necessary, and will inform the investigators of such regulatory reports. Site investigators must submit safety reports as required by their IRB. Documentation of the submission and receipt by the IRB must be retained for each safety report.

10. Type and Duration of Follow-up of Participants after ADEs

ADEs/UADEs will be followed until resolved or considered stable. Adverse events will be followed by the investigator or a clinician member of the study team in person if the participant is hospitalized for an ADE or UADE. If the participant is not hospitalized, the investigator or a clinician may review the participant’s medical record, contact the participant by phone, or contact the participant’s primary care physician for follow-up. Participant safety data will be reviewed on an ongoing basis by the sponsor.

VIII. ADMINISTRATIVE, ETHICAL, REGULATORY CONSIDERATIONS

The study will be conducted according to the Declaration of Helsinki, the US Code of Federal Regulations described for the Protection of Human Subjects (21 CFR 50), Financial Disclosure by Clinical Investigators (21 CFR 54), Institutional Review Boards (21 CFR 56), and the abbreviated Investigational Device Exemption (IDE) requirements (under 21 CFR 812.2(b)).

To maintain confidentiality, all records other than consent forms will be identified by a coded number only and only the site Principal Investigator or coordinator shall maintain an enrollment log linking coded identifiers to personally identifiable information. The Investigator must also comply with all applicable national and local privacy regulations (e.g., Health Insurance Portability and Accountability Act of 1996 [HIPAA], EU Data Protection Directive 95/46/EC) and the requirements of their reviewing IRB/IEC.

A. Informed Consent Form

Informed consent will be obtained in accordance with the Declaration of Helsinki, International Conference on Harmonization (ICH) GCP, 21 CFR 50.25, CFR 50.27, and CFR Part 56, Subpart A, HIPAA, if applicable, and local regulations.

The Sponsor will prepare informed consent forms and HIPAA authorizations for approval by the IRB/IEC. The written consent documents will embody the elements of informed consent as described in the ICH GCP Guidelines, and also comply with FDA and local regulations. IRB/IEC-approved copies of the blank Informed Consent Forms will be maintained as part of the study files.

Study investigators and qualified staff members with consenting privileges will obtain informed consent. All participants will receive a verbal explanation in terms suited to their comprehension of the purposes, procedures and potential risks of the study. The participants must have the ability to understand and sign an informed consent form, which must be signed prior to enrollment. A properly executed, written, informed consent must be obtained from each participant prior to entering the participant into the trial. Information will be given in both oral and written form and participants must be given ample opportunity to inquire about details of the study and they will be informed that they may withdraw from the study at any time without prejudice to themselves or loss of benefits. If a site is participating in the “sub-study” for repeatability and reproducibility (see Section XI), participants must “opt-in” to the sub-study by signing an additional or supplemental consent form in order to have the additional imaging performed, and may decline to be in the sub-study without affecting their participation in the main study cohort. If the participant requires the consent to be in larger font in order to read it well, this will be provided. If participants are visually impaired to the point of being unable to read the consent (but still meet inclusion criteria), they can take the consent back with them to read it over with a family member or with the use of magnifying devices. If the participant chooses, the investigator can also read the consent verbatim to the participant and answer any questions that may arise. If a participant is unable to sign the informed consent form (ICF) and the HIPAA authorization, a legal representative may sign for the participant. A copy of the signed ICF will be given to the participant or legal representative of the participant and the original will be maintained by the Investigator. The investigator obtaining consent should document the consent process in the participant’s medical record. Participants who do not understand the ICF due to language or other barriers will not be eligible for the study.

B. Participant Confidentiality

All medical records will be kept confidential and will only be reviewed by the participating investigators. In order to maintain participant confidentiality, only a site number and coded participant number will identify all study participants on CRFs and other documentation submitted to the Sponsor, FPRC or CRO, in addition to basic participant demographic information. The participants’ name will not appear on any of the images exported to the Reading Center or any of the data forms reported to the CRO. Participants’ personal information will be kept as private as possible. However, records can be inspected by organizations for quality assurance and data analysis. These include the members of the FDA, IRB and the CRO.

C. Individual Withdrawal Criteria

Participants may choose to withdraw from this study, including the repeatability and reproducibility sub-study, for any reason at any time without penalty, loss of benefits or prohibition from enrolling in other clinical protocols.

Reasons for participant discontinuation may include, but are not limited to, the following:

- Investigator determination that study continuation is not in the best medical interest of the participant;
- Findings in the course of the trial that may affect willingness to participate;

- Study eye requires ocular surgery or ocular injections which cannot safely be postponed until after the end of the study;
- Inability to keep study visits or to comply with study requirements;
- Any other safety concerns.

IX. STUDY DESIGN

This is a multi-center observational study. Study participants will be recruited at primary care sites. Evaluation of the study participant inclusion/exclusion criteria must be based on the review of clinic medical records and/or documented patient interviews conducted by the Investigator or under the direction of the Investigator using qualified study staff (e.g., nursing staff or research coordinators) trained by the Investigator in the application of the study inclusion and exclusion criteria.

A. Participant Inclusion criteria:

1. Documented diagnosis of diabetes mellitus, e.g.:
   a. Having met the criteria established by either the World Health Organization (WHO) or the American Diabetes Association (ADA)
   b. Hemoglobin A1c (HbA1c) ≥ 6.5%
   c. Fasting Plasma Glucose (FPG) ≥ 126 mg/dL (7.0 mmol/L)
   d. Oral Glucose Tolerance Test (OGTT) with two-hour plasma glucose (2-hr PG) ≥ 200 mg/dL (11.1 mmol/L), using the equivalent of an oral 75 g anhydrous glucose dose dissolved in water
   e. Symptoms of hyperglycemia or hyperglycemic crisis with a random plasma glucose (RPG) ≥ 200 mg/dL (11.1 mmol/L)
2. Age 22 or older
3. Understand the study and volunteer to sign the informed consent

B. Participant Exclusion criteria:

- Persistent vision loss, blurred vision, or floaters.
- Diagnosed with macular edema, severe non-proliferative retinopathy, proliferative retinopathy, radiation retinopathy, or retinal vein occlusion.
- History of laser treatment of the retina or injections into either eye, or any history of retinal surgery.
- Currently participating in another investigational eye study and actively receiving investigational product for DR or DME.
- Participant has a condition that, in the opinion of the investigator, would preclude participation in the study (e.g., unstable medical status including blood pressure or glycemic control, microphthalmia or previous enucleation).
- Participant is contraindicated for imaging by fundus imaging system used in the study:
  - Participant is hypersensitive to light
  - Participant recently underwent photodynamic therapy (PDT)
  - Participant is taking medication that causes photosensitivity

C. Clinical Site Selection

The coordinating center (CRO), with the agreement of the Sponsor, will select clinical sites according to their interest and ability to participate and to sign an appropriate

investigator agreement with the Sponsor, the ability of the investigational site to be reviewed and approved for this study by a qualified IRB/IEC, the presence of staffing suitable to conduct protocol-required assessments, the availability to follow this protocol and utilize other written procedures or SOPs regarding the conduct of clinical research, and with input on geographic diversity and suitable patient populations from the study Sponsor. See Qualification of the Sites below.

1. Primary Care (PC) Site inclusion criteria

- Frontline care site with an adequate patient population likely to meet the inclusion/exclusion criteria
- Ethnic and racial distribution suitable to achieve study overall ethnic and racial distribution goal (in combination with other selected sites)
- Availability of space to have ocular fundus and OCT cameras on site, including the following to be installed and maintained by the Sponsor, only for the purposes of the study
  - Topcon TRC-NW400 Non-Mydriatic Retinal Camera, accompanied by a laptop computer connected to the camera to control and inform the imaging process
  - A separate Topcon 3D OCT-1 Maestro camera system compliant with FPRC's 4-W imaging protocol and includes a Spectral Domain OCT system used for "Gold Standard" imaging
- Availability of staff to train and take fundus photographs per IDx-DR imaging protocol
- Internet connection and computer hardware (supplied) for operation of the IDx-DR device and eCRF forms
- Willing to administer tropicamide 1.0% eye drops to participants that do not provide images suitable for IDx-DR analysis with undilated eyes
- Previous experience with clinical studies preferred or demonstrated aptitude for conducting study
- Willingness to allow a FPRC-certified ophthalmic photographer onsite for "Gold Standard" reference standard imaging using the Maestro system

2. NW400 Camera Operators at Sites

All personnel who will be taking fundus images and operating the IDx-DR device will be documented and trained as per the IDx training protocol to become NW400 operators. NW400 operators must attest to the fact that they have not had previous experience operating a fundus imaging system prior to participating in this study. Training for the NW400 camera operators will consist of written materials and a standardized training session on the use of the IDx-DR Client, the image quality feedback mechanism, and operation of the NW400 fundus camera. Written materials include the fundus camera operator manual, a quick reference guide for operations, image quality, and troubleshooting, and training certification checklist. NW400 operators will self-certify as IDx-DR photographers by successfully submitting exams to IDx-DR. Following certification, these photographers will be referred to as qualified 'NW400 operators'.

D. Overview of Primary Care Enrollment & Data Collection Process

- Potential participants are first identified by the primary care physician or staff using patient history.

- Potential participants are further evaluated by a site research coordinator who determines eligibility using the inclusion and exclusion criteria.
- The research coordinator or Investigator discusses the trial and obtains informed consent. The participant is now in the participant population and is assigned an identification code for the study.
- The NW400 operator then takes the participant to the NW400 fundus camera and runs through the imaging protocol, until either the IDx-DR device has reported images of sufficient quality, or dilation is necessary to complete the collection of images suitable for analysis. If, after dilation, readable images are still not obtained, the IDx-DR exam result will be considered insufficient quality, and the study participant continues to the OCT and 4-W imaging protocols.

- Participants providing IDx-DR images of sufficient quality for reporting continue and are assessed using the OCT and 4-W imaging protocols.
- If dilation is necessary, the participant is first asked to remove contact lenses if they are wearing contacts. The NW400 operator (or other qualified site staff) under medical supervision then dilates the participant's pupils with tropicamide 1.0% topical eye drops. The participant is then asked to wait 30 minutes or until the pupils are dilated to at least 5mm. The imaging protocol is then followed again.
- If exams of sufficient quality cannot be acquired after dilation, the IDx-DR result will be logged and indicated as an insufficient exam quality result.
- The research coordinator saves the IDx-DR result and the study information about the participant is recorded and submitted to the Internet-based Electronic Data Capture (EDC) system maintained by the CRO.
- The participant is then transferred to the FPRC certified photographer. After concluding the IDx-DR imaging protocol, if the participant is not already dilated, the participant is asked to remove contact lenses if they are wearing contacts. Then tropicamide 1.0% is administered by the FPRC certified photographer and the participant is asked to wait 30 minutes or until the pupils are dilated to at least 5mm. Once the participant is dilated, FPRC certified photographer performs the following:
    - W-4 stereo four field fundus photography according to the FPRC W-4 protocol.
    - Photography for media opacity assessment
    - OCT of the macula using a standardized system capable of producing at least 121 slices, according to the FPRC OCT protocol.
- Participant is transferred to the research coordinator who then verifies the completeness of the data collected and gives the participant compensation as approved by the IRB/IEC for his or her time. If a participant did not bring their own sunglasses, the site will provide a pair of disposable sunglasses to reduce glare and possible discomfort from having dilated pupils. A supplemental compensation for local transportation may also be provided if needed and approved by the IRB/IEC. The study participant will be exited from the study at this point.

E. Masking Protocol

IDx has chosen an independent data storage center for the clinical trial. The IDx-DR software will be locked prior to initiating the clinical trial. A copy will be placed in escrow prior to the start of enrollment for auditing purposes. Prior to initiating the clinical trial, IDx will deliver access passwords to a third party, who will be instructed to change the passwords in order to prevent IDx from having direct access to the system during testing. This third party will

ensure that IDx cannot have access to the device or its backups. During trial, any maintenance activities, cybersecurity updates, or patches necessary during the course of the clinical trial will go through the third party who will only take action upon instructions from an authorized IDx representative. All activities will be logged and recorded. At the conclusion of the clinical trial, IDx will provide the final version for comparison with the initial version placed in escrow along with the activity logs. All trial data will be copied to external encrypted storage and delivered to a designated third party, after which trial servers will be securely erased by the third party so that IDx can access the servers without accessing the data.

Documentation, logging and audited record will provide evidence of two important items: 1) IDx does not have direct access to any trial data during the course of the trial, and 2) after the trial, study data will continue to be masked to IDx. It is expected that ongoing masking associated with item 2 will allow the use of study data for validation of subsequent product releases without the need for additional prospective data collection.

F. Primary Care Imaging Procedure

1. IDx-DR SCREENING PROCEDURE (Topcon TRC-NW400 Imaging Platform, by the NW400 operator)

Imaging and IDx-DR submission activities should follow the instructions included with the IDx-DR device..

2. 4-W & OCT REFERENCE STANDARD IMAGING (by the FPRC certified photographer)

1. If participant did not undergo dilation as part of the IDx-DR imaging protocol, they will be asked to remove contact lenses if they are wearing contacts and a single drop of tropicamide 1.0% will be administered in the conjunctival sac of each eye under supervision of a licensed provider and the participant is asked to wait 30 minutes.
2. Once the participant has undergone dilation, he or she will be imaged by the certified photographer according to the reference standard protocols provided by the Reading Center.
    a. W-4 stereo four field fundus photography according to the FPRC W-4 protocol.
    b. Photography for media opacity assessment
    c. OCT of the macula using a standardized system capable of producing at least 121 slices, according to the FPRC OCT protocol.
3. Once the participant has undergone reference standard photography, the PC site research coordinator verifies the completeness of the data collected and gives the participant compensation for his or her time as approved by the IRB/IEC. A supplemental compensation for local transportation may also be provided if needed and approved by the IRB/IEC.
4. The PC site research coordinator or designated FPRC photographer submits the documented OCT and W-4 images to the RC.
5. The PC site research coordinator or principal investigator maintains a copy of the signed informed consent form at the PC site.
6. The PC site research coordinator or principal investigator maintains an enrollment log with the identification code and actual identity of the participant.
7. The PC site research coordinator keeps a log of participants consented, imaged, and dilated.

X. STUDY DESIGN (ENRICHED COHORT)

At the start of the study, all participants with diabetes who meet inclusion and exclusion criteria will be enrolled. To avoid excessive enrollment in any one stratum (no/mild, mtmDR), the totals will be monitored monthly and the study population may be enriched to ensure sufficient numbers of

subjects with mtmDR. Participant enrichment will be targeted based on elevated HbA1c and/or FPG, factors which have been shown to be correlated with higher rates of mtmDR among patients with diabetes. No cap will be placed on enriched participants since such participants will be prospectively recruited.

The impact on enrollment of enrichment based on elevated HbA1c and/or FPG is not known. The goal will be to recruit enough subjects to satisfy each stratum. If enrollment patterns are not on track with the study's target population after periodic reviews, enrollment at all sites will be adjusted monthly by modifying the HbA1c and FPG, e.g., narrowing to participants with an HbA1c of 9% or higher, or a FPG in excess of 200 mg/dL. Adjustments from this level may be instituted after periodic review of the stratum enrollments reflecting the number of participants with diabetic retinopathy or diabetic macular edema. Study sites will be notified by the CRO the first work day of each month if there is to be a change in the HbA1c or FPG thresholds. Once the total number of participants with mtmDR has been projected in alignment with statistical power requirements, then the final adjustments to participant enrollment criteria will be performed.

Monitoring of strata will be performed by the study statistician (without access to IDx-DR results); only available HbA1c and FPG, along with FPRC categorization into mDR and vtDR, will be visible for each subject included to date. The study statistician will notify the CRO before the first of each month whether updated HbA1c and/or FPG thresholds are needed. Again, once the strata targets for mtmDR and no/mild DR can be projected, the HbA1c and FPG thresholds will be locked.

A. Participant Inclusion criteria for enriched cohort:

1. Diagnosis of diabetes mellitus (as defined above in Section IX.A, except as follows): HbA1c and/or FPG level as defined by statistician during enrichment period
2. Age 22 or older
3. Understand the study and volunteer to sign the informed consent

B. Participant Exclusion criteria:

- Persistent vision loss, blurred vision, or floaters.
- Diagnosed with macular edema, severe non-proliferative retinopathy, proliferative retinopathy, radiation retinopathy, or retinal vein occlusion.
- History of laser treatment of the retina or injections into either eye, or any history of retinal surgery.
- Currently participating in another investigational eye study and actively receiving investigational product for DR or DME.
- Participant has a condition that, in the opinion of the investigator, would preclude participation in the study (e.g., unstable medical status including blood pressure or glycemic control, microphthalmia or previous enucleation).
- Participant is contraindicated for imaging by fundus imaging system used in the study:
    - Participant is hypersensitive to light
    - Participant recently underwent photodynamic therapy (PDT)
    - Participant is taking medication that causes photosensitivity

C. Clinical Site Selection

All sites will participate in enrichment, if implemented, and will be willing to administer HbA1c or FPG tests on participants to determine eligibility for enrollment, if the participant's medical record lacks adequate documentation to establish the diagnosis of diabetes meeting an enrichment criteria, or for de novo patients being screened for study inclusion.

D. Primary Care Imaging Procedure

Other than site selection and HbA1c/FPG testing, the site qualification and imaging workflow will be identical to that of the prospective cohort.

XI. READING CENTER GRADING PROTOCOL

A Grading and Imaging Charter has been established to define image grading processes associated with the University of Wisconsin Fundus Photography Reading Center's generation of a "gold standard".

Participant Identification Codes
All data and forms will carry a unique identification code. This code will consist of the study site number and the participant number at that site. The codes will not reveal the participant's name, any personal health information about the participant, or any indication of the IDx-DR result.

Certification
The Reading Center will certify any ophthalmic photographers where photographers have not previously been certified. The Reading Center may grandfather existing certifications if candidates are currently certified for similar procedures in other studies and have been actively performing the procedures.

Timeline (Estimated Dates)
The period for conduct of the study is expected to last 7 months, beginning in 2017.

XII. APPENDIX VI: REFERENCES

1. Klonoff, D.C. and D.M. Schwartz, *An economic analysis of interventions for diabetes.* Diabetes Care, 2000. **23**(3): p. 390-404.
2. CDC. *Vision Health Initiative*. 2015 [cited 2015 August 4, 2015]; Available from: http://www.cdc.gov/visionhealth/basic_information/eye_disorders.htm#a5.
3. Bressler, N.M., et al., *Underuse of the health care system by persons with diabetes mellitus and diabetic macular edema in the United States.* JAMA Ophthalmol, 2014. **132**(2): p. 168-73.
4. *Early photocoagulation for diabetic retinopathy. ETDRS report number 9. Early Treatment Diabetic Retinopathy Study Research Group.* Ophthalmology, 1991. **98**(5 Suppl): p. 766-785.
5. *Photocoagulation for diabetic macular edema. Early Treatment Diabetic Retinopathy Study report number 1. Early Treatment Diabetic Retinopathy Study research group.* Arch.Ophthalmol., 1985. **103**(12): p. 1796-1806.
6. Diabetic Retinopathy Study, G., *Photocoagulation treatment of proliferative diabetic retinopathy: clinical application of DRS findings: DRS report 8.* Ophthalmology, 1981. **88**: p. 583-600.
7. Diabetic Retinopathy Study, G., *Indications for photocoagulation treatment of diabetic retinopathy: DRS report 14.* Int Ophthalmol Clin, 1987. **27**: p. 239-253.

8. Helmchen, L.A., H.P. Lehmann, and M.D. Abramoff, *Automated Detection of Retinal Disease.* Am J Manag Care, 2014. **11**(17).
9. Abramoff, M.D., et al., *Automated analysis of retinal images for detection of referable diabetic retinopathy.* JAMA Ophthalmol, 2013. **131**(3): p. 351-7.
10. National Institute of Diabetes and Digestive and Kidney Diseases. *What is the Current State of Quality of Care in Diabetes?* 2013 [cited 2015 02]; Available from: http://www.ncqa.org/PublicationsProducts/OtherProducts/QualityProfiles/FocusonDiabetes/WhatistheCurrentStateofQualityofCare.aspx.
11. Hazin, R., M.K. Barazi, and M. Summerfield, *Challenges to establishing nationwide diabetic retinopathy screening programs.* Curr Opin Ophthalmol, 2011. **22**(3): p. 174-9.
12. Lee, D.J., et al., *Dilated eye examination screening guideline compliance among patients with diabetes without a diabetic retinopathy diagnosis: the role of geographic access.* BMJ Open Diabetes Res Care, 2014. **2**(1): p. e000031.
13. Liew, G., M. Michaelides, and C. Bunce, *A comparison of the causes of blindness certifications in England and Wales in working age adults (16-64 years), 1999-2000 with 2009-2010.* BMJ Open, 2014. **4**(2): p. e004015.
14. Mansberger, S.L., et al., *Long-term Comparative Effectiveness of Telemedicine in Providing Diabetic Retinopathy Screening Examinations: A Randomized Clinical Trial.* JAMA Ophthalmol, 2015.
15. Sim, D.A., et al., *Automated retinal image analysis for diabetic retinopathy in telemedicine.* Curr Diab Rep, 2015. **15**(3): p. 14.
16. American Diabetes Association. *Statistics About Diabetes*. 2014 [cited 2016 February 16, 2016]; Available from: http://www.diabetes.org/diabetes-basics/statistics/?referrer=https://www.google.com/.
17. Bresnick, G.H., et al., *A screening approach to the surveillance of patients with diabetes for the presence of vision-threatening retinopathy.* Ophthalmology, 2000. **107**(1): p. 19-24.
18. Centers for Disease Control and Prevention. *Age-Adjusted Percentage of Adults Aged 18 Years or Older with Diagnosed Diabetes Receiving a Dilated Eye Exam in the Last Year, United States, 1994–2010*. 2014; Available from: http://www.cdc.gov/diabetes/statistics/preventive/fX_eye.htm.
19. Hazin, R., et al., *Revisiting diabetes 2000: challenges in establishing nationwide diabetic retinopathy prevention programs.* Am J Ophthalmology, 2011. **152**(5): p. 723-9.
20. Inan, U.U., F. Ozturk, and S.S. Ermis, *Pharmacologic pupil dilation in diabetic patients.* Retina, 2003. **23**(2): p. 254-6.
