# Supplementary material for: Pivotal trial of an autonomous AI-based diagnostic system for detection of diabetic retinopathy in primary care offices
Source: NPJ Digit Med. 2018 Aug 28;1:39. doi: 10.1038/s41746-018-0040-6 (PMC6550188; doi:10.1038/s41746-018-0040-6)
Supplement: Supplementary file 3 — Supplemental Tables 1 and 2 [file 41746_2018_40_MOESM3_ESM.docx]

Supplemental Tables

| **Worst ETDRS Level** | **Count** | **Frequency** |
| --- | --- | --- |
| 10 | 382 | 0.466 |
| 12 | 83 | 0.101 |
| 14A | 0 | 0.000 |
| 14B | 5 | 0.006 |
| 14C | 0 | 0.000 |
| 14Z | 0 | 0.000 |
| 15 | 18 | 0.022 |
| 20 | 133 | 0.162 |
| 35A | 0 | 0.000 |
| 35B | 2 | 0.002 |
| 35C | 46 | 0.056 |
| 35D | 18 | 0.022 |
| 35E | 15 | 0.018 |
| 35F | 56 | 0.068 |
| 43A | 14 | 0.017 |
| 43B | 20 | 0.024 |
| 47A | 8 | 0.010 |
| 47B | 3 | 0.004 |
| 47C | 0 | 0.000 |
| 47D | 0 | 0.000 |
| 53A | 0 | 0.000 |
| 53B | 0 | 0.000 |
| 53C | 0 | 0.000 |
| 53D | 1 | 0.001 |
| 53E | 0 | 0.000 |
| 60 | 5 | 0.006 |
| 61A | 0 | 0.000 |
| 61B | 5 | 0.006 |
| 65A | 2 | 0.002 |
| 65B | 1 | 0.001 |
| 65C | 1 | 0.001 |
| 71A | 0 | 0.000 |
| 71B | 1 | 0.001 |
| 71C | 0 | 0.000 |
| 71D | 0 | 0.000 |
| 75 | 0 | 0.000 |
| 81 | 0 | 0.000 |
| 85A | 0 | 0.000 |
| 85B | 0 | 0.000 |

**Supplemental Table 1a**

| **Worst Eye DME** | **Count** | **Frequency** |
| --- | --- | --- |
| Clinically significant DME (‘fundus’) | 29 | 0.035 |
| Center-involved DME (‘OCT’) | 19 | 0.023 |
| Clinically significant DME AND/OR  Center involved DME | 42 | 0.051 |

**Supplemental Table 1b**

**Supplemental Table 1**. Prevalence of Diabetic Retinopathy in the study by 1a) ETDRS severity level of the worst eye; 1b) DME level of the worst eye. Frequencies are calculated based on the subset of 819 analyzable participants.

| **Worst ETDRS Level** | **Description** | **Fundus-based**  **Clinically Significant Macular Edema** | **Fundus based classification** |
| --- | --- | --- | --- |
| 10 | No or Mild NPDR | absent | mtmDR- |
| 12, 14A, 14B, 14C,14Z, 15, 20 | Mild NPDR |  |  |
| 10 | No or Mild NPDR | a) zone of retinal thickening > 1 disc area, part < 1 disc diameter from foveal center  b) retinal thickening or adjacent hard exudates < 600µm from foveal center | mtmDR+ |
| 12, 14A, 14B, 14C, 14Z, 15, 20 | Mild NPDR |  |  |
| 35A, 35B, 35C, 35D, 35E, 35F, 43A, 43B, 47A, 47B, 47C, 47D | Moderate NPDR | absent or present | mtmDR+ |
| 53A, 53B, 53C, 53D, 53E | Severe NPDR | absent or present | mtmDR+  vtDR+ |
| 60, 61A, 61B, 65A, 65B, 65C, 71A, 71B, 71C, 71D, 75, 81, 85A, 85B | PDR |  |  |

**Supplemental Table 2.** Mapping of Early Treatment of Diabetic Retinopathy Study (ETDRS) severity levels (worst eye) and combination with fundus based Diabetic Macular Edema (DME) to the dichotomous categories *fundus mtmDR* – (no or mild NPDR and no DME),
*fundus mtmDR* + (more than moderate DR: more than mild DR or DME), as well as *vtDR*+ (vision threatening DR: severe NPDR, or PDR, or DME). NPDR = Non Proliferative DR.
